# Cytokinin-dependent secondary growth determines root biomass in radish (*Raphanus sativus* L.)

**DOI:** 10.1093/jxb/erv220

**Published:** 2015-05-15

**Authors:** Geupil Jang, Jung-Hun Lee, Khushboo Rastogi, Suhyoung Park, Sang-Hun Oh, Ji-Young Lee

**Affiliations:** ^1^School of Biological Sciences, College of Natural Science, Seoul National University, Seoul 151-747, Korea; ^2^Department of Horticultural Crop Research, National Institute of Horticultural and Herbal Science, Wanju 565-852, Korea; ^3^Department of Biology, Daejeon University, Daejeon 300-716, Korea

**Keywords:** Cambium, cytokinin, *Raphanus sativus*, root biomass, secondary growth.

## Abstract

The root serves as an essential organ in plant growth by taking up nutrients and water from the soil and supporting the rest of the plant body. Some plant species utilize roots as storage organs. Sweet potatoes (*Ipomoea batatas*), cassava (*Manihot esculenta*), and radish (*Raphanus sativus*), for example, are important root crops. However, how their root growth is regulated remains unknown. In this study, we characterized the relationship between cambium and radial root growth in radish. Through a comparative analysis with *Arabidopsis* root expression data, we identified putative cambium-enriched transcription factors in radish and analysed their expression in representative inbred lines featuring distinctive radial growth. We found that cell proliferation activities in the cambium positively correlated with radial growth and final yields of radish roots. Expression analysis of candidate transcription factor genes revealed that some genes are differentially expressed between inbred lines and that the difference is due to the distinct cytokinin response. Taken together, we have demonstrated for the first time, to the best of our knowledge, that cytokinin-dependent radial growth plays a key role in the yields of root crops.

## Highlight

Comparative studies using *Arabidopsis* and radish (*Raphanus sativus*) found that cytokinin-mediated regulatory programmes in the cambium are important for the radial growth of radish roots and its variations.

## Introduction

Vascular plants are characterized by their dynamic and indeterminate growth in both apical and radial directions. Apical (primary) and lateral (secondary) meristems are responsible for such development. Cambium, a secondary meristem, drives the radial growth in stems and roots. Undifferentiated cells in the cambium undergo asymmetric cell divisions in anticlinal or periclinal directions to generate daughter cells that become a part of the xylem or phloem tissues. Several environmental components such as temperature, photoperiod, and precipitation affect the secondary growth driven by the cambium (Antonova and Stasova, 1997; Begum *et al.*, 2013).

Secondary growth is important for biomass production. Therefore, there has been a growing interest in understanding the physiological regulation underlying secondary growth (Miyashima *et al.*, 2013; Zhang *et al.*, 2014). Auxin and cytokinin are key regulators in this. Auxin is distributed differentially across the cambial regions in hybrid aspen; it reaches a maximum level in cambium regions and then decreases in regions forming tracheids (Uggla *et al.*, 1996; Uggla *et al.*, 1998). Changes in auxin distribution seem to affect the orientation of cambial initials in response to wounding (Kramer *et al.*, 2008). However, the initiation of latewood formation and the cessation of cambial cell division are not caused by changes in auxin concentration in the cambium of Scots pine (Uggla *et al.*, 2001), indicating the involvement of other plant hormones in these processes. Recently, cytokinins were shown to regulate the cambium activity and secondary growth. When four genes encoding isopentenyl transferase (*IPT1*, -*3*, -*5*, and -*7*) were disrupted in *Arabidopsis*, cambium formation became defective and root thickening was reduced (Matsumoto-Kitano *et al.*, 2008). A study by Nieminen *et al.* (2008) also demonstrated the essential role of cytokinin in *Populus* cambium. They showed that cytokinin receptor genes are preferentially expressed in the dividing cambial cells in the *Populus* stem. Overexpression of a gene encoding CYTOKININ OXIDASE (CKX), a cytokinin-degrading enzyme, resulted in the suppression of secondary growth (Nieminen *et al.*, 2008).

Gene regulatory networks governed by transcription factors are essential for every aspect of plant development. High-resolution transcript profiling has revealed genes that are expressed in a cambium-enriched manner in *Populus* (Schrader *et al.*, 2004). However, only a small number of transcription factors have been identified as regulators of secondary growth so far. These include *Arabidopsis thaliana Homeo-Box8* (*ATHB8*), *Populus REVOLUTA*, *High Cambial Activity2* (*HCA2*), *Populus LATERAL ORGAN BOUNDARIES DOMAIN 1* (*PtaLBD1*), and *Ethylene Response Factor 109* (*ERF109*) and *ERF18* (Baima *et al.*, 2001; Guo *et al.*, 2009; Yordanov *et al.*, 2010; Robischon *et al.*, 2011; Etchells *et al.*, 2012).

Radial growth occurs not only in stems but also in roots. Root radial growth is particularly noticeable in storage roots, many of which serve as important sources of food and energy. Anatomical studies have shown that storage roots form cambium as well as anomalous meristematic tissues, and their organization varies depending on plant species (Esau, 1977). However, the more detailed nature of storage root growth at structural and molecular levels remains to be elucidated. Radish (*Raphanus sativus*) belonging to the Brassicaceae is an economically important root crop in Eastern Asia. It has advantages over other root crops for the study of radial root growth for following reasons. First, radish root growth is very rapid, completing its major growth within 9 weeks. Secondly, radish is accessible for tissue-specific gene expression analysis because of its thick root morphology. Lastly, cross-species comparison with a model species is relatively straightforward due to the evolutionary proximity of radish to *Arabidopsis thaliana*, whose root development has been studied extensively. Currently, draft radish genome sequence data (Kitashiba *et al.*, 2014) and about 300 000 expressed sequence tags (ESTs) are publicly available (Shen *et al.*, 2013).

In this study, we characterized the radial growth in radish roots, and found that the cambial cell division tightly correlated with the radial root growth. We selected 11 transcription factor genes whose expression was highly enriched in *Arabidopsis* root cambium and examined expression patterns of their putative orthologues in radish roots. We discovered some radish transcription factors that are highly enriched in the root cambium in a developmental stage-dependent manner. Further characterization of expression patterns revealed that these genes were differentially expressed between inbred lines showing distinctive radial root growth and that the difference was connected to the distinct cytokinin responses in the cambium zones of the inbred lines. Taken together, our study suggests that the regulation of cambial cell division plays an essential role in radial root growth, and that cytokinin and its downstream transcription factors contribute to this process as key components.

## Materials and methods

### Plant materials, growth, and phenotypic analysis

The radish inbred lines used in this study were obtained from National Institute of Horticultural and Herbal Science (NIHHS) of the Republic of Korea. Each inbred line was produced by manually self-pollinating F2s between two cultivars, ‘Kwan-dong summer’ and ‘Pyeong-ji summer’, for 10 generations. Phenotypic analysis was carried out for the radish inbred lines grown in the field at NIHHS, Suwon (127′01″E/37′16″N), Korea. For growing radish in a growth room, seeds were germinated in pots (20×20×20cm) filled with soil and grown at 22°C, under a 16h light/8h dark photoperiod. Root circumference was measured at the thickest part of a root. Photoshop and Image J were used for processing radish images and measuring roots and shoots.

### Embedding, sectioning, and staining

For transverse sectioning of radish roots, specimens (0.5×0.5×0.5cm) collected from the thickest parts of radish roots were fixed overnight in 4% paraformaldehyde dissolved in PBS (pH 7.4). After washing with PBS, fixed samples were dehydrated in an increasing concentration of ethanol (25, 50, 75, and 100%) in PBS and then in a series of Neo-Clear mixed with 100% ethanol (25, 50, 75, and 100%). The dehydrated samples were sequentially incubated in an increasing series of paraffin concentrations [25, 50, 75, and 100%, v/v, in Neo-Clear (Merck)] for 1 d each time. Samples were then incubated in 100% paraffin for 2 d and then placed in moulds. Solidified samples were sectioned at a thickness of 8–10 µm with a RM 2145 microtome (Leica). Deparaffinized and hydrated sections were stained with 0.05% toluidine blue (pH 4.4). Images were captured with an axioimager M1 (Zeiss) and IX70 (Olympus) light microscope system.

### Immunolocalization assay

To visually analyse cell division activity in cambium tissues, immunolocalization of radish roots was carried out with proliferating cell nuclear antigen (PCNA) antibody (Santa Cruz Biotechnology). The thickest part of the root was transversely sectioned with a razor blade and fixed overnight in 4% paraformaldehyde dissolved in PBS at 4 °C. The root sections were then washed with PBS buffer six times for 10min each. They were pre-incubated with 2% BSA in PBS for 30min and then with PCNA antibody diluted at a ratio of 1:100, for 1.5h at 37 °C and again washed with PBS, six times for 10min each. The root sections were incubated with Alexa Fluor 488-conjugated anti-goat IgG (Invitrogen), diluted 200-fold in PBS for 1h at room temperature and washed six times in PBS for 10min each, and then stained with propidium iodide and mounted on a slide glass with citifluor (Electron Microscopy). The fluorescence signals from the Alexa Fluor 488 were detected using an LSM700 confocal microscope (Zeiss). The excitation/emission wavelength was 488nm and 505–530nm for Alexa Fluor 488 and 561 and 591–635nm for propidium iodide.

### Identification of cambium-enriched transcription factor orthologues in radish and *Arabidopsis*

Transcription factors that are expressed in a cambium-enriched manner were identified by analysing the data, which combined the root expression map (Brady *et al.*, 2007) and the new expression data generated for early procambium/cambium. Specifically, *Arabidopsis* plants expressing *ARR15::erGFP* [containing endoplasmic reticulum-targeted green fluorescent protein (erGFP)], which indicates early cambium, and plants expressing *ARR5::erGFP*, which indicates procambium and root cap were developed. Expression profiles for these cell types were generated using a method used for other root expression data based on protoplast sorting-microarray technology (Birnbaum *et al.*, 2005; Lee, J.-Y., Nieminen, K., Elo, A., Weissmann, S. and Helariutta, Y., unpublished data). A manuscript about more details of these data is in preparation. In the combined root expression data, approximately 170 transcription factors were identified to be enriched in the early cambium from the search for co-expressed gene groups (modules) (Segal *et al.* 2003). For this study, 11 transcription factors were selected based on their enriched expression in the early cambium and procambium as well as their potential importance as meristem regulators, such as ANT and KNAT1.

The radish coding sequence (CDS) and EST sequence data were collected from RGD (ftp://ftp.kazusa.or.jp/pub/radish/) and RadishBase (http://bioinfo.bti.cornell.edu/cgi-bin/radish/index.cgi). Orthologous genes were searched for based on the reciprocal BLAST analysis (cut-off value of 1E^–4^). The top hit for both blasts (*Arabidopsis* CDS against radish CDS/EST and radish CDS/EST against *Arabidopsis* CDS) were selected and considered as putative orthologous gene pairs.

BLASTP analysis for the *Arabidopsis* CDS and its homologues as well as radish orthologue candidates was carried out. Alignments of assembled nucleotide sequences were conducted using TranslatorX, an online tool that aligns nucleotide sequences based on the translated amino acid sequences (Abascal *et al.*, 2010). Poorly aligned regions were removed from tree analysis either manually or using default parameters in TranslatorX. The phylogenetic bootstrap analyses were conducted for the nucleotide sequence data of each gene-family data using maximum parsimony in the program PAUP* version 4.0b10 (Swofford, 2003). Bootstrap analysis (Felsenstein 1985) with 500 pseudoreplicates was conducted. For options of maximum parsimony analysis, we used simple sequence addition and tree bisection and reconnection branch swapping to find the best tree in each replication.

### Cytokinin treatment

To analyse the short-term effect of cytokinin on line 216 and 218 root cambia, root cubes (0.5×0.5×0.5cm) were collected from the thickest parts of line 216 and 218 root grown in growth room conditions for 5 weeks, treated with MS liquid medium containing 20 µM 6-benzylaminopurine (BAP), and then incubated for 3h at room temperature. For gene expression analysis in response to cytokinin treatment, total RNAs were extracted from cambium regions isolated from these root cubes. To analyse the long-term effect of cytokinin on the root growth, 2 l of water containing 20 or 200nM of BAP was fed to 3-week-old radish plants every alternative day for a week.

### Quantitative reverse transcription PCR (qRT-PCR) analysis

To analyse temporal and spatial expression patterns of candidate genes, qRT-PCR analyses were carried out using total RNAs extracted from root tissues, which were collected at various developmental stages. Cambial zone and inner parenchyma regions 1 and 2, which are located at one-third and two-thirds of the distance from the cambium zone to the root centre, were collected. Cambial zones can be visually distinguished from neighbouring tissues because of their high cell density. These regions were carefully thin sectioned and collected. Total RNA extraction was performed with an RNeasy Plant Mini-prep kit according to the manufacturer’s instructions (Qiagen). The reverse transcription reaction (20 µl) was performed for the first cDNA strand synthesis using 1 µg of total RNA and Superscript III reverse transcriptase (Invitrogen). After completion of the reverse transcription reaction, the cDNA template was diluted 5-fold by adding 80 µl of ddH_2_O, and 1 µl of cDNA template was used for the quantitative PCR in a total volume of 10 µl. For quantitative PCR, a master mix was prepared using an iQTM SYBR Green supermix (Bio-Rad) and PCR was carried out according to the manufacturer’s instructions (initial denaturation at 95 °C for 3min, followed by 40 cycles of denaturation at 95 °C for 10 s, annealing at 57 °C for 10 s and extension at 72 °C for 30 s). PCR and fluorescence detection were performed using a CFX96 Real-Time PCR machine (Bio-Rad), and three technical replicates of the qRT-PCRs were performed using two biological replicates. Primer information is available in Supplementary Table S1, available at *JXB* online. *RsActin2/7* was used as an internal control gene for the analysis of candidate gene expression, and the stability of *RsActin2/7* as a reference gene was tested by another internal control gene, *Radish Translation elongation factor 2* (*RsTEF2*) (Xu *et al.*, 2012). Expression levels obtained from real-time RT-PCR were calculated by subtracting the cycle threshold (*C*_t_) values of each target gene from the *C*_t_ values of *RsActin2/7*. Expression values obtained for each tissue at different time points or in different root regions were normalized by row and then displayed as heat maps using the Multi-Experiment Viewer (MeV), which is part of the TM4 microarray software suite (Saeed *et al.*, 2006).

## Results

### Root yield depends on the radial root growth in radish

**Fig. 1.**
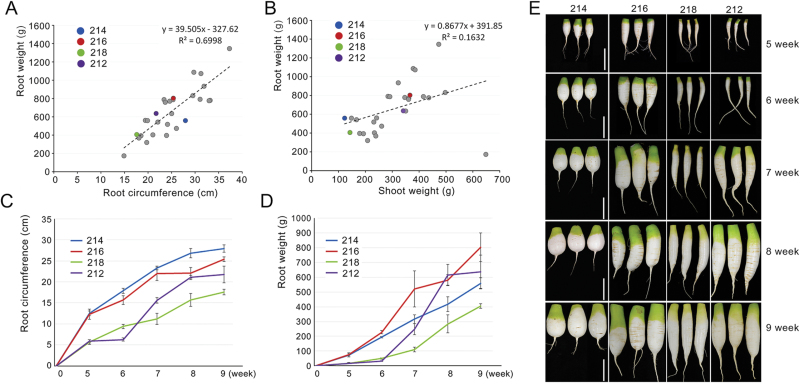
Characterization of radial root growth patterns in radish inbred lines. (A, B) Scatter plots showing relationship between radish growth factors: root weight and root circumference (A), and root weight and shoot weight (B). Dotted lines indicate trend lines. (C, D) Different root growth patterns of selected inbred lines. Root growth patterns of the indicated inbred lines were analysed by measuring root circumference (C) and root weight (D) at the indicated times (*n≥*4). (E) Morphological changes in roots of selected inbred lines. Bars, 10cm.

As the first step towards understanding the radial root growth in radish, we analysed relationships between radial root growth and other growth parameters. We measured root fresh weight, root circumference, shoot fresh weight, and shoot length in 28 radish inbred lines grown in the field for 9 weeks (Fig. 1A, B, and Supplementary Fig. S1, available at *JXB* online). When average root circumferences for inbred lines were plotted against average root weights, a strong positive correlation (*R* ^2^=0.70) was found between root weights and root circumferences (Fig. 1A). This indicates that radial root growth is an important factor determining root yield in radish. However, radish root growth was not tightly linked to the shoot growth. When average shoot weights or shoot lengths were plotted against root weights, the correlation was much weaker (R^2^ =0.16 for shoot weight and 0.07 for shoot length) than the correlation between root weight and root circumference (Fig. 1B and Supplementary Fig. S1A). The poor correlation between root and shoot growth was also found when shoot weights and lengths were plotted against root circumferences (*R* ^2^=0.1 for shoot weight and 0.03 for shoot length) (Supplementary Fig. S1B, C).

To further investigate the radial root growth in radish, we chose four inbred lines (lines 212, 214, 216, and 218) and characterized their radial root growth patterns over time (Fig. 1C–E, and Supplementary Fig. S2, available at *JXB* online). Lines 214 and 216 were selected because their radial growth in 5 weeks was superior to the growth of other inbred lines tested. Lines 212 and 218 were selected as representatives of poor radial growth. During the growth period from 5 to 9 weeks after seed planting, lines 214 and 216 showed consistently active radial growth, resulting in higher root biomass than lines 212 and 218. Even though line 212 was selected because of its poor radial growth over 5 weeks, it showed accelerated growth between 6 and 8 weeks. By contrast, the radial root growth of line 218 was very slow throughout the entire growth period, resulting in the lowest root biomass. Despite its poor radial root growth, the shoot growth of line 218 plants was similar to that of line 214 at all developmental stages. The poor correlation between root and shoot growth was also observed in the comparison between line 212 and other lines (Supplementary Fig. S2). Taken together, these results suggested that root-specific developmental mechanisms control radial root growth and final root yields.

### Radial root growth accompanies dynamic changes in the cambial zone

Cambium is the stem-cell population that drives radial growth. Asymmetric cell division in the cambial zone generates cells for xylem and phloem tissues in the stem (Nieminen *et al.*, 2008; Zhang *et al.*, 2011). In *Populus*, the number of thin cell layers in a stem cambial zone increases during growth seasons and then decreases in winter. It is known that such seasonal changes in the size of the stem cambial zone are due to changes in the relative rates between cell division and cell differentiation.

**Fig. 2.**
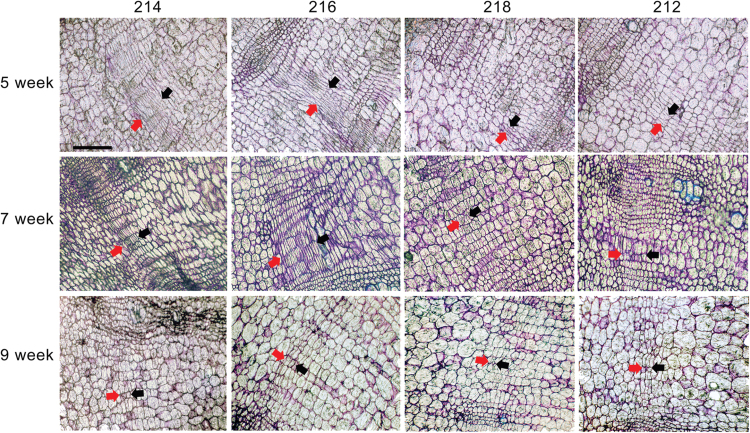
Dynamic changes in the cambial organization of radish roots. Cross-section images of root cambia in radish inbred lines. Developmental stage-dependent changes in root cambium were captured by imaging transverse sections stained with toluidine blue, which were prepared from 5-, 7-, and 9-week-old radish plants. The cambium zone is indicated with red and black arrows. The red arrows are on the side of the cortex region. Bars, 100 μm.

Similar to the radial growth in the stem, the cambium might function as a key location that drives radial growth in the root. In this case, we would expect that the cambial zones would show structural differences in radish inbred lines that have varied growth dynamics. To test our hypothesis, we searched for cambium-like cell populations in radish roots that would be organized similar to the stem cambium. Time-course images obtained by free-hand transverse sectioning showed the presence of opaque areas with high cell densities that lined up parallel to the epidermis (Supplementary Fig. S3, available at *JXB* online). To analyse these areas in detail, we generated thin sections from paraffin-embedded root blocks and stained them with toluidine blue. The stained sections revealed that opaque regions are composed of layers of thin cells that are stacked in varying numbers (Fig. 2). These cellular organizations are typical of cambium zones in the stem. Inbred lines showing active radial growth such as lines 214 and 216 displayed dynamic changes in cambial zones over time (Fig. 2). In lines 214 and 216, the cambial zones of 5- and 7-week-old roots expanded to around 10 cell layers, while those in 9-week-old roots shrank to about two cell layers. In contrast to lines 214 and 216, dynamic changes in the cambial zone were less discernible in line 218 whose cambium had only two to five thin cells at all developmental stages. The size of the cambial zones in line 212 was between that seen in lines 214 and 216 and in line 218.

**Fig. 3.**
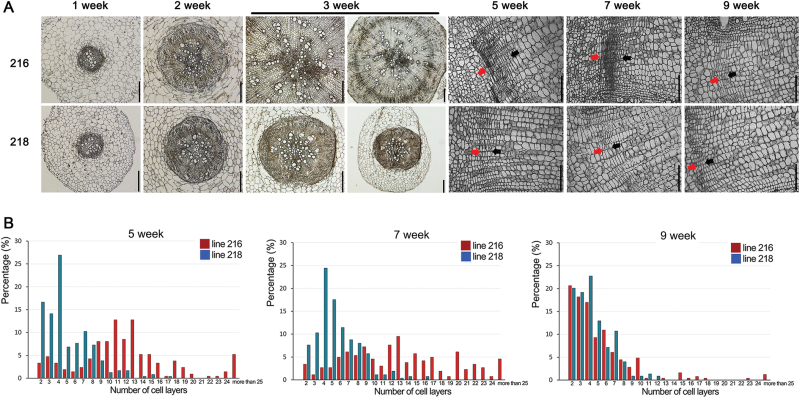
Distinct cambium development in line 216 and 218 roots. (A) Trace of time-dependent changes in root cambium development of lines 216 and 218. Cambium zones are indicated by red and black arrows with the red arrows on the side of the cortex region. (B) Quantification of cambial cell layers in 5-, 7-, and 9-week-old roots of lines 216 and 218 (*n*>210). Bars, 200 μm (1- to 3-week-old roots); 100 μm (5- to 9-week-old roots).

More detailed analyses of the cambial cell layers were performed for lines 216 and 218, which showed contrasting radial growth. This time we grew the plants in a growth room. Radish root growth in growth room conditions was almost identical to that in the field, indicating that our growth room conditions could successfully replicate the field conditions (Supplementary Fig. S4, available at *JXB* online). First, we imaged thin cross-sections of roots from 1-, 2-, and 3 week-old plants (Fig. 3A). At 1 week after germination, lines 216 and 218 had already started establishing cambium tissue, as indicated by the dense cell layers formed between the xylem and ground tissues. At 2 weeks after germination, we observed a further increase in the number of xylem vessels and neighbouring parenchyma cells. Up until this stage, no noticeable difference in cellular organization was found between lines 216 and 218. However, in 3-week-old plants, a dramatic increase in cell numbers inside the cambial layer was observed in line 216. By contrast, line 218 showed only a slight increase. These results suggested that the difference in radial growth of lines 216 and 218 was from differences in the cell proliferation activities in the cambium. Next, we counted the cambial cell layers in various regions of cross-sections and compared their distributions (Fig. 3A, B) in 5-, 7-, and 9-week-old roots. At 5 and 7 weeks, when the radial growth was active for line 216, significantly more cambial cell layers were present in line 216 than in line 218 (Supplementary Table S2, available at *JXB* online). However, such differences between lines 216 and 218 diminished in 9-week-old plants.

**Fig. 4.**
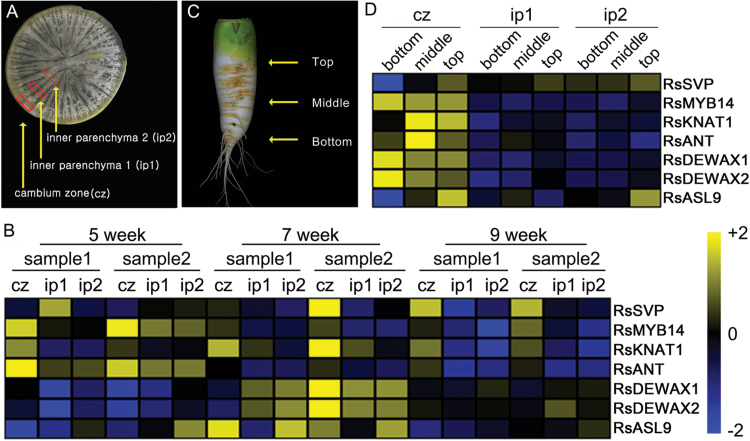
Developmental stage-dependent regulation of cambium-enriched transcription factor genes in radish. (A) Image showing the cambium zone and inner parenchyma regions 1 and 2 used for the analysis of candidate gene expression. (B) Heat map showing developmental stage-dependent changes in cambium-enriched transcription factor genes in line 216 plants. (C) Image showing different longitudinal tissues used for the analysis of candidate gene expression. (D) Heat map showing cambium-enriched expression patterns of the candidate genes in different longitudinal positions. Gene expression values were calculated by subtracting target *C*_t_ values from control *C*_t_ and then normalized by each row.

It has been reported that ectopic meristems develop and contribute to the growth of root crops (Esau, 1977). We analysed this aspect further by imaging cellular organization in the cambium region, inner parenchyma region 1, inner parenchyma region 2, and root centre of line 216 roots grown for 7 weeks (Fig. 4A and Supplementary Fig. S5, available at *JXB* online). As reported previously, several regions filled with small cells were found, which indicated the presence of ectopic meristems. The density of ectopic meristems was slightly higher in the root centre than in the regions closer to the cambium. In cambial zones of actively growing stems and roots, cells emerging from the cambium by asymmetric cell division gradually increase their sizes to differentiate. By contrast, cells adjacent to putative ectopic meristems were fully expanded, indicating that these cells were not recent descendants of ectopic meristems. These results further suggested that the cambium might be primarily responsible for the radial growth of radish roots.

### Cell division activities in the cambium drive radial root growth

**Fig. 5.**
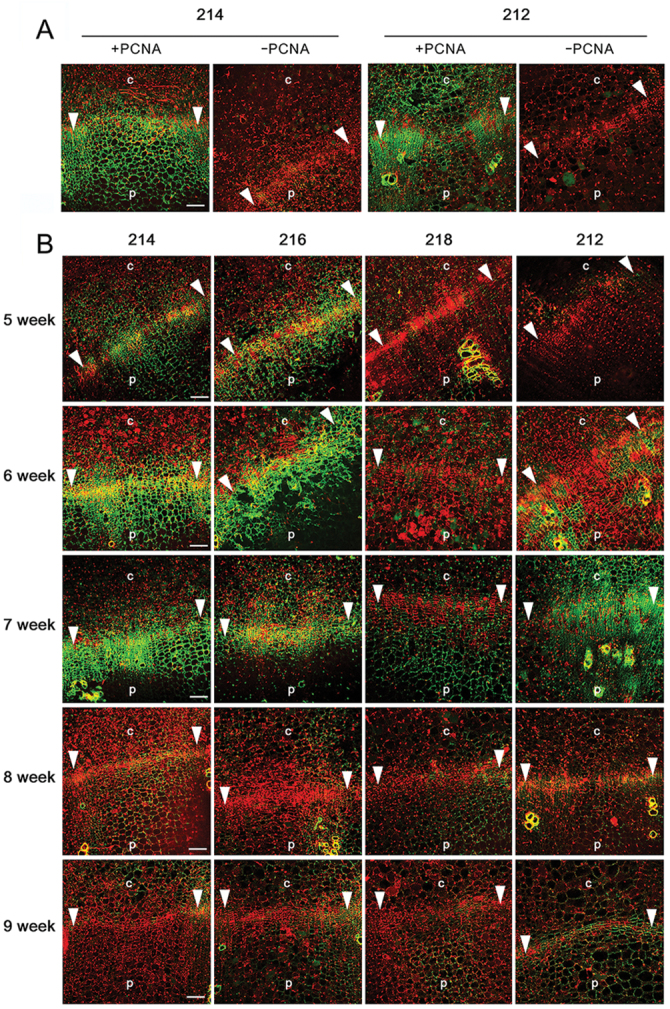
Dynamic changes in the cambial activity of radish roots. (A) Immunolocalization of PCNA in radish roots. PCNA expression was analysed in 7-week-old roots of lines 212 and 214 by immunolocalization assays, with (+) and () indicate PCNA antibody-treated and untreated conditions, respectively. (B) Developmental stage-dependent regulation of cambial activity. The cambial activity of the indicated inbred lines was analysed by immunolocalization of PCNA over time. c, Cortex; p, parenchyma; arrowhead, cambium. Bars, 200 μm.

To understand the relationships between dynamic changes in the cambial zone and cell division activity we performed immunolocalization assays in the cambial zones using an antibody against PCNA. PCNA is an evolutionarily conserved protein that is required for DNA replication. Its expression is highly upregulated in actively dividing cells (Dimova *et al.*, 2003; Shimizu and Mori, 1998a, *b*). Our test experiment using 7-week-old root sections of lines 212 and 214 with or without treatment with PCNA antibody confirmed that the dividing cell-specific fluorescence signals came only from antibody-treated sections (Fig. 5A). High expression of PCNA in root cambium was also confirmed in actively growing scarlet globe radish roots (Supplementary Fig. S6, available at *JXB* online). We then carried out immunolocalization analyses using the roots of lines 212, 214, 216, and 218 collected at five different developmental stages (Fig. 5B). Line 214 and 216 plants showed a high level of PCNA in the cambium zone between 5 and 7 weeks, and then a very low level at 8 and 9 weeks. Line 212 plants did not show PCNA expression in 5-week-old roots but showed increasing PCNA levels in the cambium zone of 6-week-old roots. PCNA expression reached its highest level in 7-week-old root and then disappeared abruptly in line 212. In contrast to lines 212, 214, and 216, PCNA expression was always below the detection level in line 218. This indicated that the cambial cell division activity of line 218 plants was very low compared with the other inbred lines. Such developmental stage-dependent changes in cambial cell division together with dynamic changes in cambium size and cell organization in inbred lines suggest that cell division activities in the cambium play a critical role in the radial growth of radish roots.

### Cross-species comparison identifies cambium-enriched transcription factors in radish

Cambium structure and its physiological regulation have been characterized in stems of woody eudicots (Savidge, 1996; Antonova and Stasova, 1997; Lachaud *et al.*, 1999). However, underlying genetic components and molecular mechanisms are still largely unknown. Regulatory networks by transcription factors are crucial for every aspect in plant development, and cambium development and growth driven by cambial activities are no exception to this.

To understand transcriptional regulation in the radish root cambium, we searched radish ESTs and predicted CDSs that are putatively orthologous to cambium-enriched transcription factors in *Arabidopsis* roots. Genes for cambium-enriched transcription factors in *Arabidopsis* roots were identified from previous root cell-type-specific transcript profiling data combined with expression profiles in the early stage of root cambium formation (Birnbaum *et al.*, 2003; Nawy *et al.*, 2005; Lee *et al.*, 2006; Levesque *et al.*, 2006; Brady *et al.*, 2007; Carlsbecker *et al.*, 2010) (Supplementary Fig. S7, available at *JXB* online). These include *SHORT VEGETATIVE PHASE* (*SVP*, *AT2G22540*), *KNOTTED-LIKE FROM ARABIDOPSIS THALIANA* (*KNAT1*, *AT4G08150*), and *AINTEGUMENTA* (*ANT*) (*AT4G37750*). SVP, a flowering time regulator like SUPPRESSOR OF OVEREXPRESSION OF CONSTANS 1 (SOC1) and FRUITFULL (FUL), showed strong enriched expression in the early cambium. SOC1 and FUL have been shown to regulate the meristem determinacy, thereby affecting secondary growth (Melzer *et al.*, 2008). KNAT1 and ANT (AINTEGUMENTA), key regulators in the shoot apical meristem, also show strong enrichment in the early cambium. KNAT1 was recently shown to affect the xylem cell fate in the cambium (Liebsch *et al.*, 2014). Through reciprocal BLAST analyses between *Arabidopsis* and radish, we identified 12 putative orthologues from radish (Table 1). Orthologous relationships between *Arabidopsis* and radish gene pairs were further confirmed by phylogenetic tree analyses (Supplementary Fig. S8, available at *JXB* online).

**Table 1.** *Putative cambium-enriched transcription factors in radish, selected based on* Arabidopsis *root expression map*

*Arabidopsis* gene name	TF motif	Gene name of radish orthologue	Radish EST	Putative radish CDS
At2g22540 (SVP)	MADS	RsSVP	FY439133	Rsa1.0_01930.1_g00004.1
At2g31180 (MYB14)	MYB	RsMYB14	EY908024	Rsa1.0_00496.1_g00007.1, Rsa1.0_03050.1_g00002.1
At4g08150 (KNAT1)	Homeobox	RsKNAT1	EV532705	Rsa1.0_00263.1_g00002.1
At4g37750 (ANT)	AP2	RsANT	EY917101	Rsa1.0_00792.1_g00008.1
At5g61590 (DEWAX)	ERF	RsDEWAX-1	EV545694	–
		RsDEWAX-2	EV528492	Rsa1.0_00056.1_g00002.1
At1g16530 (ASL9)	ASL	RsASL9	–	Rsa1.0_00065.1_g00008.1
At3g23250 (MYB15)	MYB	RsMYB15	FY440652	Rsa1.0_01399.1_g00001.1, Rsa1.0_02960.1_g00001.1
At2g38470 (WRKY33)	WRKY	RsWRKY33	FY448049	Rsa1.0_03437.1_g00004.1
At5g51190 (ERF105)	ERF	RsERF105	FY434566	Rsa1.0_07250.1_g00002.1
At1g17380 (JAZ5)	JAZ	RsJAZ5	FD981520	Rsa1.0_00153.1_g00014.1
At2g47260 (WRKY23)	WRKY	RsWRKY23	FY443167	Rsa1.0_01588.1_g00008.1

To analyse the candidate gene expression patterns during radial root growth, we extracted total RNA from the cambium region and inner parenchyma regions 1 and 2 in 5-, 7- and 9-week-old roots from line 216 and analysed changes in gene expression by tracing their relative expression against *RsActin2/7*, a radish reference gene for qRT-PCR (Fig. 4A, B). To validate *RsActin2/7* stability as a reference, we tested the relative expression of another radish reference gene, *RsTEF2*, against *RsActin2/7* (Xu *et al.*, 2012). The relative expression ratios between *RsTEF2* and *RsActin2/7* were very stable in all tissues of 5- and 7-week-old line 216 roots; however, some fluctuation in *RsTEF2* was found in 9-week-old roots (Supplementary Fig. S9, available at *JXB* online). Thus, we chose *RsActin2/7* as our primary reference gene. Among the 12 selected genes, seven exhibited a tendency to be expressed in the cambium region more highly than in other two regions (Fig. 4B), while the other five genes showed barely any enriched expression in the cambium (Supplementary Fig. S10, available at *JXB* online). Thus, we further analysed the seven cambium-enriched genes in more detail, and detected a dynamic nature in their expression patterns. For example, cambium-enriched expression of *RsKNAT1* was consistently detected at all developmental stages. However, the other genes showed developmental stage-dependent regulation. Expression of *RsSVP* in 5-week-old root cambium was similar to or slightly lower than that in the inner parenchyma tissues, but it became higher in the cambium than in the inner parenchyma tissues in 7- and 9-week-old roots. *RsMYB14* and *RsANT* showed much stronger enrichment in 5-week-old root cambium than in 7- and 9-week-old root cambium. In contrast to these results, *RsDEWAX1*, *RsDEWAX2*, and *RsASL9* reached their maximum expression in 7-week-old roots.

To further understand the expression patterns of the cambium-enriched genes, we extracted total RNA from the cambium region and inner parenchyma regions 1 and 2 at three different longitudinal axes of 7-week-old roots of line 216 and analysed gene expression (Fig. 4C, D). As expected, all the candidates were expressed in the cambium region more highly than in the other two tissues. Among these, expression of *RsSVP*, *RsKNAT1*, *RsANT*, and *RsASL9* in the cambium changed dynamically along the longitudinal axis. Taking these data together, we concluded that expression of transcription factors in the cambium is regulated in a very dynamic manner over space and time.

### Differential expression of cambium-enriched transcription factors in inbred lines with distinctive radial root growth

**Fig. 6.**
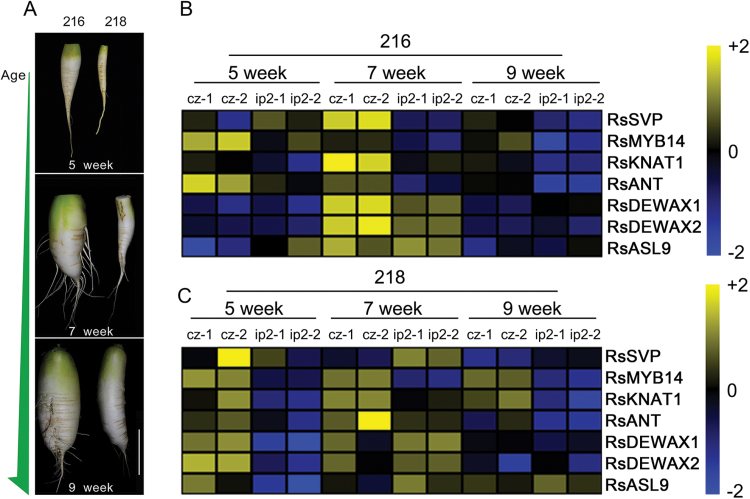
Expression patterns of cambium-enriched genes in two radish inbred lines with distinctive root growth. (A) Root morphology of line 216 and 218 plants grown in growth room condition for 5, 7, and 9 weeks. (B) Heat map showing developmental stage-dependent expression patterns of the candidate genes in line 216 root cambium. (C) Heat map showing developmental stage-dependent expression patterns of the candidate genes in line 218 root cambium. Gene expression values were calculated by subtracting target *C*_t_ from control *C*_t_ values and then normalized by each row. Bars, 10cm.

Line 218 plants showed suppressed radial root growth compared with line 216 plants. This suggested that expression patterns of the cambium-enriched genes might be different in these two. To test this idea, we analysed the mRNA levels of the aforementioned cambium-enriched genes in 216 and 218 roots. qRT-PCR was performed using total RNA extracted from the cambium zone and inner parenchyma region 2 of lines 216 and 218 roots grown in growth room conditions for 5, 7, and 9 weeks (Fig. 6 and Supplementary Fig. S11, available at *JXB* online). Interestingly, the expression pattern of *RsSVP* in line 216 roots was different from those in line 218. As mentioned in the previous section, line 216 plants showed dynamic regulation of *RsSVP* in cambium regions along root development stages. By contrast, the stage-dependent enrichment of *RsSVP* in the cambium was not observed in line 218 roots. Expression patterns of *RsDEWAX1* and *RsDEWAX2* also showed striking differences between line 216 and 218 plants. *RsDEWAX1*, *RsDEWAX2*, and *RsASL9* did not show strong cambium-enriched expression in 5-week-old root cambium of line 216, although their expression increased dramatically in 7-week-old roots. By contrast, line 218 displayed very strong cambium-enriched expression of *RsDEWAX1*, *RsDEWAX2*, and *RsASL9* in 5-week-old roots. Expression patterns of other genes such as *RsMYB14*, *RsKNAT2*, and *RsANT* were similar between line 216 and 218 roots (Supplementary Fig. S11). Despite some differences in expression levels (Fig. 6B), expression of these genes was highly enriched in the root cambium throughout all stages in both lines. These results suggested that *RsSVP*, *RsDEWAX1*, *RsDEWAX2*, and *RsASL9* are involved in the cambium-mediated radial growth.

### Differences in radial growth caused by cytokinin responsiveness in radish

**Fig. 7.**
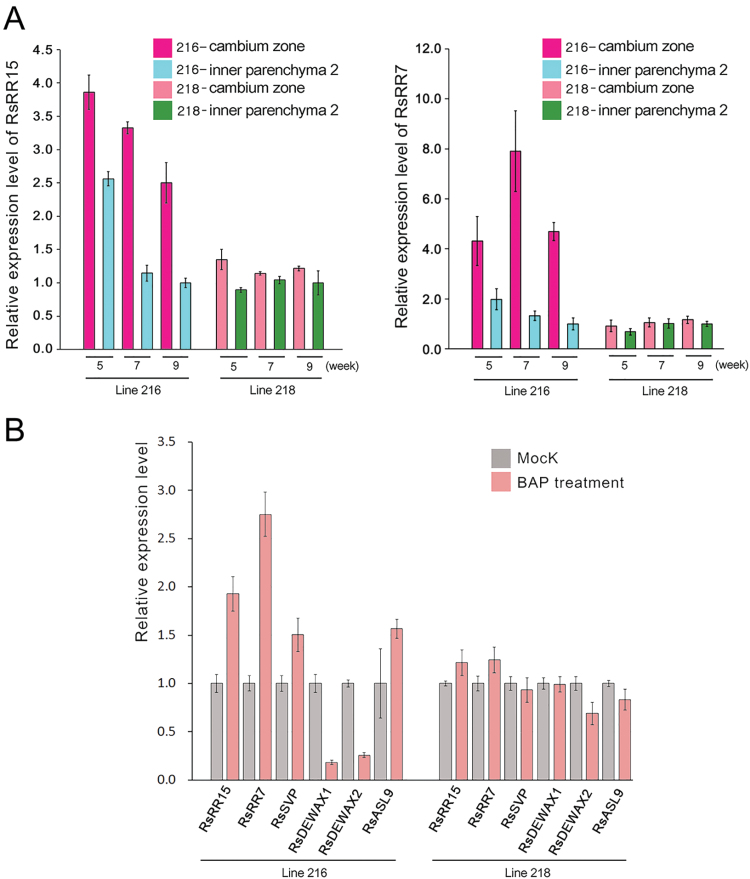
Radish inbred lines with distinctive root growth show differences in cytokinin responses. (A) Expression analysis of *RsRR15* and *RsRR7* as indicators of the cytokinin response. Expression of *RsRR15* and *RsRR7* was analysed by qRT-PCR using total RNA extracted from the cambium zone and inner parenchyma region 2 of lines 216 and 218 at three different developmental stages. Gene expression in inner parenchyma region 2 of 9-week-old plants was used as the standard for analysing expression changes. (B) Expression analysis of cytokinin-responsive genes in lines 216 and 218. Expression of *RsRR15*, *RsRR7*, and candidate genes was analysed in 20 μM BAP-treated and untreated root cambium of 5-week-old plants by qRT-PCR. Gene expression in the untreated mock was used as the standard for analysing expression changes.

Previously, it was shown that the expression patterns of *SVP*, *DEWAX*, and *ASL9* are regulated by cytokinin (Naito *et al.*, 2007; Mantiri *et al.*, 2008; Bhargava *et al.*, 2013). Cytokinin is a plant hormone essential in maintaining cell division activity and secondary growth. Because *RsSVP*, *RsDEWAX1*, *RsDEWAX2*, and *RsASL9* showed distinctive expression patterns in lines 216 and 218, we asked whether cytokinin status was different between these two lines. To address this, we traced changes in cytokinin responses in line 216 and 218 root cambium by analysing the expression levels of *RsRR15* and *RsRR7*, radish orthologues of *Arabidopsis Response Regulator 15* (*ARR15*) and *ARR7* (Fig. 7A). In line 216 roots, the expression levels of *RsRR15* and *RsRR*7 were always higher in the cambium than in the inner parenchyma. In contrast to line 216, cambium enrichment of *RsRR15* and *RsRR*7 was weak in line 218 roots. Furthermore, developmental stage-dependent changes in *RsRR15* and *RsRR*7 expression in the root cambium were not observed in line 218. These results indicated that the response of line 218 to cytokinin in the root cambium might be weaker than the response of line 216. To further understand the cytokinin response in line 216 and 218 root cambium, we compared cytokinin responses induced by exogenous cytokinin treatment in these two inbred lines (Fig. 7B). Cytokinin treatment of line 216 roots induced the expression of *RsRR15* and *RsRR*7 in the root cambium by 2- and 2.7-fold, respectively, in comparison with untreated root cambium. By contrast, *RsRR15* and *RsRR*7 in the root cambium of line 218 were induced only slightly by cytokinin. Other genes known to be affected by cytokinin also showed expression changes in line 216 but not in line 218. *RsSVP* and *RsASL9* were induced in line 216 but not in line 218, and *RsDEWAX1* and *RsDEWAX2* were significantly repressed by cytokinin in line 216 but not in line 218. These results suggested that the weak cytokinin response of line 218 cambium might be caused by defects in the cytokinin signalling and not by cytokinin biosynthesis.

**Fig. 8.**
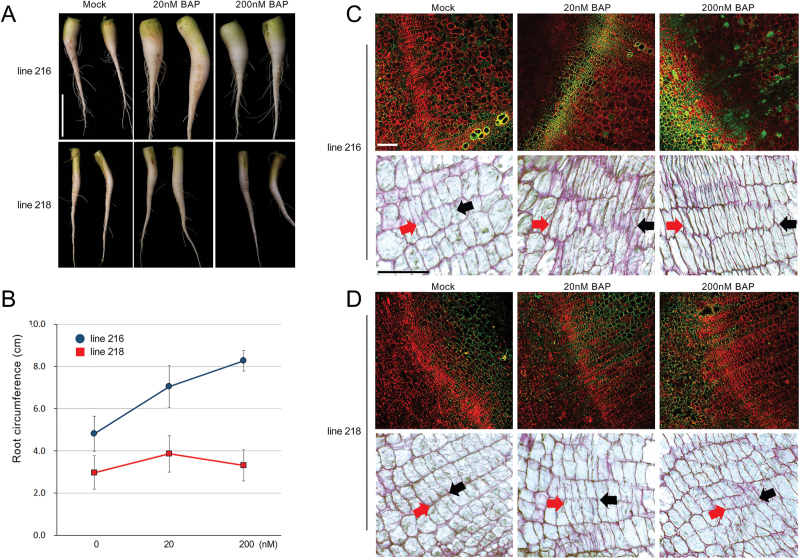
Secondary root growth in radish is directly affected by cytokinin signalling. (A) Morphological changes in line 216 and 218 roots in response to cytokinin treatment. (B) Relationship between cytokinin and secondary root growth. (*n*≥3). (C, D) Cytokinin effect on cambium activity and structure in line 216 (C) and 218 (D) plants. Cambium activity was analysed by immunolocalization of PCNA (top panels), and cambium structure was observed by staining transverse sections with toluidine blue (bottom panels). The cambium zone is indicated with red and black arrows. The red arrows are on the side of the cortex region. Bars, 5cm (A); 200 μm (immunolocalization images of C and D); 100 μm (transverse section images of C and D).

If line 218 plants were defective in cytokinin signalling, their radial root growth would not be affected by cytokinin treatment. To test this hypothesis, we analysed the effect of exogenous cytokinin on root growth in lines 216 and 218 (Fig. 8A, B). When 3-week-old plants in line 216 were grown in the cytokinin-treated condition for 1 week, their roots became noticeably thicker than untreated roots. In addition, their root growth showed dependence on cytokinin dosage; 20 and 200nM BAP induced an increase in root circumferences of 50 and 75%, respectively, compared with the mock treatment. In contrast to line 216, line 218 plants did not display cytokinin dosage-dependent activation of radial root growth; 20nM BAP induced only a slight increase in root circumference, while 200nM BAP resulted in roots very similar to untreated roots. We then observed cambial cell division activity and cambium structure in response to cytokinin (Fig. 8C, D). When cell division activities in the cambium were monitored by immunolocalization of PCNA, line 216 roots grown without exogenous cytokinin did not show active cell division in the cambial area. By contrast, those treated with 20 and 200nM BAP showed a dramatic increase in cell division activity in the cambium zone in a dosage-dependent manner. Consistent with the change in cell division activities, cell organization in the cambium of line 216 was also affected by cytokinin. Cytokinin treatment induced an increase in the cambium size. In contrast to line 216 plants, line 218 plants did not respond to cytokinin. Cambial cell division activity and the cambium structure of line 218 roots treated with cytokinin were almost identical to the mock-treated control plant. These results demonstrated that the suppressed radial growth of line 218 roots was caused by defects in cytokinin signalling. Therefore, cytokinin signalling and its downstream transcriptional regulation play an essential role in the radial growth of radish roots.

## Discussion

The root is an essential organ required for nutrient and water uptake from soil. A growing number of studies show that plant growth and yields are affected by root development, suggesting that modulation and optimization of root development are crucial for the next green revolution (Villordon *et al.*, 2014). In radish, secondary growth seems to be directly linked to root yield. Previous structural analyses showed the formation of both cambium and internal anomalous meristematic tissues during radial root growth in radish (Esau, 1977). However, it has been unclear to what extent the cambium and anomalous meristems contribute to the growth. In this study, we found that the cambial activity is strongly correlated with radial root growth and biomass in radish, not with anomalous meristems, indicating the essential role of cambium in radial root growth.

Cambial activity is sensitively modulated by environmental conditions, but its molecular mechanisms are still largely unknown. Cytokinin has been considered an important signalling molecule for this process because it controls cell division activity. Cytokinin levels are also sensitively regulated by environmental signals: they decrease in water-deficit conditions but increase in nutrient-rich conditions (Samuelson *et al.*, 1992; Takei *et al.*, 2001; Yang *et al.*, 2001). Recent studies have demonstrated that cytokinin signalling is tightly linked to the regulation of secondary growth in the stem. Both quadruple mutant plants that lack *AtIPT1*, *3*, -*5*, and -*7* in *Arabidopsis* and transgenic plants overexpressing cytokinin oxidase in *Populus* showed suppressed secondary growth (Matsumoto-Kitano *et al.*, 2008; Nieminen *et al.*, 2008). The proliferative role of cytokinin has also been suggested based on the studies of root meristem in *Arabidopsis* (Mähönen *et al.*, 2000; Dello Ioio *et al.*, 2007). In mutants defective in cytokinin signalling such as *wooden leg* (*wol*) and *ahk2 ahk3 ahk4* triple-mutant plants, the cell division that increases the number of procambial cell files is suppressed, resulting in the reduction of cell files that constitute primary vascular tissues (Scheres *et al.*, 1995; Mähönen *et al.*, 2006).

Previous studies by Radin and Loomis (1971) and Webster and Radin (1972) suggested the possible involvement of cytokinin in the secondary growth of radish roots. In these studies, they showed that cytokinin levels increased in radish roots with the onset of active secondary growth, and that cytokinin treatment triggered an increase in root diameter (Radin and Loomis, 1971; Webster and Radin, 1972). However, how cytokinin regulates this process at the molecular level has never been demonstrated. Here, we showed that cytokinin functions as a key modulator driving secondary growth and that cytokinin controls the secondary growth by regulating cell proliferation in the root cambium. In the inbred line with active secondary growth, the cytokinin response was stronger in the root cambium than in other neighbouring tissues, and was dynamically regulated along developmental stages. By contrast, the inbred line with suppressed secondary growth exhibited a very weak cytokinin response in the root cambium. Consistently, we could not find developmental stage-dependent changes in the cytokinin response in this line. Furthermore, exogenous cytokinin treatment could not activate cell division in the cambial zone and the secondary root growth in the inbred lines with suppressed growth. These results collectively indicate that the suppression of radial root growth in this inbred line is caused by defects in cytokinin signalling. However, we do not rule out the possibility that a key regulator of periclinal cell division is missing or defective in this line. Although further investigation needs to clarify these aspects, our investigation suggests that cytokinin function in secondary root growth resembles its function in secondary stem growth and that the proliferative role of cytokinin is essential for the secondary growth in radish roots.

Transcription factors are involved in every aspect of plant development by governing gene regulatory networks. Through a comparative analysis approach using *Arabidopsis* root expression data, we successfully identified cambium-enriched transcription factors in radish. Expression of *RsKNAT1* was consistently enriched in the cambium regardless of root development stage. On the other hand, genes such as *RsSVP*, *RsDEWAX1*, *RsDEWAX2*, and *RsASL9* showed developmental stage-dependent enrichment in the cambium. More importantly, the expression patterns of these genes were very different in two inbred lines with distinctive secondary root growth. Cambium-enriched expression of *RsSVP* was observed only in the inbred lines with active secondary growth, while *RsDEWAX1* and *RsDEWAX2* were expressed very strongly only in the cambium of the inbred line with suppressed secondary growth. These results showed the potential involvement of *RsSVP*, *RsDEWAX1*, *RsDEWAX2*, and *RsASL9* in secondary growth of radish roots. Previous microarray approaches using *Arabidopsis* seedlings showed that these genes are regulated by cytokinin (Bhargava *et al.*, 2013); however, their developmental functions in the cambium have not been reported. Our results uncover previously unidentified roles of RsSVP, RsDEWAX1, RsDEWAX2, and RsASL9, which are to regulate secondary root growth by controlling cytokinin-dependent cambium activity in an evolutionarily conserved manner.

A draft genome sequence of radish has become available (Kitashiba *et al.*, 2014). More than 60 000 genes are predicted, even though the assembled genome sequence is estimated to cover only 75% of the whole radish genome. Therefore, it is possible that many of radial growth regulators have evolved specifically in the radish lineage, despite the key regulators conserved to operate in both radish and *Arabidopsis*. This study shows that such evolutionarily conserved radial growth regulators can be found efficiently by comparison of radish and *Arabidopsis* data. With technical advances in genomics tool, more thorough genome-wide investigations of root development in radish will help to deepen our understandings of molecular mechanisms underlying secondary growth and to apply them to the breeding programmes of economically important root crops.

## Supplementary data

Supplementary data are available at *JXB* online.

Supplementary Table S1. Primers used in this study.

Supplementary Fig. S1. Relationships between radish growth factors.

Supplementary Fig. S2. Shoot growth pattern in selected radish inbred lines.

Supplementary Fig. S3. Images of root cross-sections in selected radish inbred lines.

Supplementary Fig. S4. Radish root development in growth room and field conditions.

Supplementary Fig. S5. Internal anatomy of radish roots.

Supplementary Fig. S6. Cell division activities in root cambia.

Supplementary Fig. S7. Cambium-enriched transcription factor genes in *Arabidopsis*.

Supplementary Fig. S8. Phylogenetic analyses of cambium-enriched transcription factor genes in *Arabidopsis* and their putative radish orthologues.

Supplementary Fig. S9. Validation of *Actin2*/*7* expression as a reference gene for qRT-PCR analysis in radish.

Supplementary Fig. S10. Expression patterns of the radish candidate genes.

Supplementary Fig. S11. Distinct expression pattern of the radish candidate genes between lines 216 and 218.

Supplementary Data

## Abbreviations:

BAP: 6-benzylaminopurine
CDS: coding sequence
EST: expressed sequence tag
PCNA: proliferating cell nuclear antigen
qRT-PCR: quantitative reverse transcription PCR.

## References

[CIT0001] Abascal F Zardoya R Telford MJ 2010 TranslatorX: multiple alignment of nucleotide sequences guided by amino acid translations. Nucleic Acids Research 38, W7–W13. 20435676 10.1093/nar/gkq291 PMC2896173

[CIT0002] Antonova GF Stasova VV 1997 Effects of environmental factors on wood formation in larch (*Larix sibirica* Ldb.) stems. Trees 11, 462–468.

[CIT0003] Baima S Possenti M Matteucci A Wisman E Altamura MM Ruberti I Morelli G 2001 The *Arabidopsis* ATHB-8 HD-zip protein acts as a differentiation-promoting transcription factor of the vascular meristems. Plant Physiology 126, 643–655. 11402194 10.1104/pp.126.2.643 PMC111156

[CIT0004] Begum S Nakaba S Yamagishi Y Oribe Y Funada R 2013 Regulation of cambial activity in relation to environmental conditions: understanding the role of temperature in wood formation of trees. Physiologia Plantarum 147, 46–54. 22680337 10.1111/j.1399-3054.2012.01663.x

[CIT0005] Bhargava A Clabaugh I To JP Maxwell BB Chiang Y-H Schaller GE Loraine A Kieber JJ 2013 Identification of cytokinin-responsive genes using microarray meta-analysis and RNA-Seq in *Arabidopsis* . Plant Physiology 162, 272–294. 23524861 10.1104/pp.113.217026 PMC3641208

[CIT0006] Birnbaum K Jung JW Wang JY Lambert GM Hirst JA Galbraith DW Benfey PN 2005 Cell type-specific expression profiling in plants via cell sorting of protoplasts from fluorescent reporter lines. Nature *Methods* 2, 615–619. 16170893 10.1038/nmeth0805-615

[CIT0007] Birnbaum K Shasha DE Wang JY Jung JW Lambert GM Galbraith DW Benfey PN 2003 A gene expression map of the *Arabidopsis* root. Science 302, 1956–1960. 14671301 10.1126/science.1090022

[CIT0008] Brady SM Orlando DA Lee J-Y Wang JY Koch J Dinneny JR Mace D Ohler U Benfey PN 2007 A high-resolution root spatiotemporal map reveals dominant expression patterns. Science 318, 801–806. 17975066 10.1126/science.1146265

[CIT0009] Carlsbecker A Lee J-Y Roberts CJ 2010 Cell signalling by microRNA165/6 directs gene dose-dependent root cell fate. Nature 465, 316–321. 20410882 10.1038/nature08977 PMC2967782

[CIT0010] Dello Ioio R Linhares FS Scacchi E Casamitjana-Martinez E Heidstra R Costantino P Sabatini S 2007 Cytokinins determine *Arabidopsis* root-meristem size by controlling cell differentiation. Current Biology 17, 678–682. 17363254 10.1016/j.cub.2007.02.047

[CIT0011] Dimova DK Stevaux O Frolov MV Dyson NJ 2003 Cell cycle-dependent and cell cycle-independent control of transcription by the *Drosophila* E2F/RB pathway. Genes and Development 17, 2308–2320. 12975318 10.1101/gad.1116703 PMC196467

[CIT0012] Esau K 1977 Anatomy of seed plants , Wiley New York, USA.

[CIT0013] Etchells JP Provost CM Turner SR 2012 Plant vascular cell division is maintained by an interaction between PXY and ethylene signalling. PLoS Genetics 8, e1002997. 23166504 10.1371/journal.pgen.1002997 PMC3499249

[CIT0014] Felsenstein J 1985 Confidence limits on phylogenies: an approach using the bootstrap. Evolution 39, 783–791. 10.1111/j.1558-5646.1985.tb00420.x 28561359

[CIT0015] Guo Y Qin G Gu H Qu L-J 2009 Dof5. 6/HCA2, a Dof transcription factor gene, regulates interfascicular cambium formation and vascular tissue development in *Arabidopsis* . The Plant Cell 21, 3518–3534. 19915089 10.1105/tpc.108.064139 PMC2798324

[CIT0016] Kitashiba H Li F Hirakawa H 2014 Draft sequences of the radish (*Raphanus sativus* L.) genome. DNA Research 21, 481–490. 24848699 10.1093/dnares/dsu014 PMC4195494

[CIT0017] Kramer EM Lewandowski M Beri S Bernard J Borkowski M Borkowski MH Burchfield LA Mathisen B Normanly J 2008 Auxin gradients are associated with polarity changes in trees. Science 320, 1610–1610. 18566279 10.1126/science.1156130

[CIT0018] Lachaud S Catesson A-M Bonnemain J-L 1999 Structure and functions of the vascular cambium. Comptes Rendus de l’Academie des Sciences, Serie III. Sciences de la Vie/Life Sciences 322, 633–650. 10505236 10.1016/s0764-4469(99)80103-6

[CIT0019] Lee J-Y Colinas J Wang JY Mace D Ohler U Benfey PN 2006 Transcriptional and posttranscriptional regulation of transcription factor expression in *Arabidopsis* roots. Proceedings of the National Academy of Sciences, USA 103, 6055–6060. 10.1073/pnas.0510607103 PMC2111400 16581911

[CIT0020] Levesque MP Vernoux T Busch W 2006 Whole-genome analysis of the SHORT-ROOT developmental pathway in *Arabidopsis* . PLoS Biology 4, e143. 16640459 10.1371/journal.pbio.0040143 PMC1450008

[CIT0021] Liebsch D Sunaryo W Holmlund M 2014 Class I KNOX transcription factors promote differentiation of cambial derivatives into xylem fibers in the *Arabidopsis* hypocotyl. Development 141, 4311–4319. 25371365 10.1242/dev.111369

[CIT0022] Mähönen AP Bishopp A Higuchi M Nieminen KM Kinoshita K Törmäkangas K Ikeda Y Oka A Kakimoto T Helariutta Y 2006 Cytokinin signaling and its inhibitor AHP6 regulate cell fate during vascular development. Science 311, 94–98. 16400151 10.1126/science.1118875

[CIT0023] Mähönen AP Bonke M Kauppinen L Riikonen M Benfey PN Helariutta Y 2000 A novel two-component hybrid molecule regulates vascular morphogenesis of the *Arabidopsis* root. Genes and Development 14, 2938–2943. 11114883 10.1101/gad.189200 PMC317089

[CIT0024] Mantiri FR Kurdyukov S Lohar DP Sharopova N Saeed NA Wang X-D VandenBosch KA Rose RJ 2008 The transcription factor MtSERF1 of the ERF subfamily identified by transcriptional profiling is required for somatic embryogenesis induced by auxin plus cytokinin in *Medicago truncatula* . Plant Physiology 146, 1622–1636. 18235037 10.1104/pp.107.110379 PMC2287338

[CIT0025] Matsumoto-Kitano M Kusumoto T Tarkowski P Kinoshita-Tsujimura K Václavíková K Miyawaki K Kakimoto T 2008 Cytokinins are central regulators of cambial activity. Proceedings of the National Academy of Sciences, USA 105, 20027–20031. 10.1073/pnas.0805619105 PMC2605004 19074290

[CIT0026] Melzer S Lens F Gennen J Vanneste S Rohde A Beeckman T 2008 Flowering-time genes modulate meristem determinacy and growth form in *Arabidopsis thaliana* . Nature Genetics 40, 1489–1492. 18997783 10.1038/ng.253

[CIT0027] Miyashima S Sebastian J Lee JY Helariutta Y 2013 Stem cell function during plant vascular development. EMBO Journal 32, 178–193. 23169537 10.1038/emboj.2012.301 PMC3553377

[CIT0028] Naito T Yamashino T Kiba T Koizumi N Kojima M Sakakibara H Mizuno T 2007 A link between cytokinin and *ASL9* (*ASYMMETRIC LEAVES 2 LIKE 9*) that belongs to the *AS2*/*LOB* (*LATERAL ORGAN BOUNDARIES*) family genes in *Arabidopsis thaliana* . Bioscience, Biotechnology, and Biochemistry 71, 1269–1278. 10.1271/bbb.60681 17485849

[CIT0029] Nawy T Lee J-Y Colinas J Wang JY Thongrod SC Malamy JE Birnbaum K Benfey PN 2005 Transcriptional profile of the *Arabidopsis* root quiescent center. The Plant Cell 17, 1908–1925. 15937229 10.1105/tpc.105.031724 PMC1167541

[CIT0030] Nieminen K Immanen J Laxell M 2008 Cytokinin signaling regulates cambial development in poplar. Proceedings of the National Academy of Sciences, USA 105, 20032–20037. 10.1073/pnas.0805617106 PMC2604918 19064928

[CIT0031] Radin J Loomis R 1971 Changes in the cytokinins of radish roots during maturation. Physiologia Plantarum 25, 240–244.

[CIT0032] Robischon M Du J Miura E Groover A 2011 The *Populus* class III HD ZIP, *popREVOLUTA*, influences cambium initiation and patterning of woody stems. Plant Physiology 155, 1214–1225. 21205615 10.1104/pp.110.167007 PMC3046580

[CIT0033] Saeed AI Bhagabati NK Braisted JC Liang W Sharov V Howe EA Li J Thiagarajan M White JA Quackenbush J 2006 TM4 Microarray Software Suite. Methods in Enzymology 411, 134–193. 16939790 10.1016/S0076-6879(06)11009-5

[CIT0034] Samuelson ME Eliasson L Larsson C-M 1992 Nitrate-regulated growth and cytokinin responses in seminal roots of barley. Plant Physiology 98, 309–315. 16668629 10.1104/pp.98.1.309 PMC1080184

[CIT0035] Savidge RA 1996 Xylogenesis, genetic and environmental regulation—a review. IAWA Journal 17, 269–310.

[CIT0036] Scheres B Di Laurenzio L Willemsen V Hauser M-T Janmaat K Weisbeek P Benfey PN 1995 Mutations affecting the radial organisation of the *Arabidopsis* root display specific defects throughout the embryonic axis. Development 121, 53–62.

[CIT0037] Schrader J Nilsson J Mellerowicz E Berglund A Nilsson P Hertzberg M Sandberg G 2004 A high-resolution transcript profile across the wood-forming meristem of poplar identifies potential regulators of cambial stem cell identity. The Plant Cell 16, 2278–2292. 15316113 10.1105/tpc.104.024190 PMC520933

[CIT0038] Segal E Shapira M Regev A Pe’er D Botstein D Friedman N 2003 Module networks: identifying regulatory modules and their condition specific regulators from gene expression data. Nature Genetics 34, 166–176. 12740579 10.1038/ng1165

[CIT0039] Shen D Sun H Huang M Zheng Y Qiu Y Li X Fei Z 2013 Comprehensive analysis of expressed sequence tags from cultivated and wild radish (*Raphanus* spp.). BMC Genomics 14, 721. 24144082 10.1186/1471-2164-14-721 PMC3816612

[CIT0040] Shimizu S Mori H 1998 *a* Analysis of cycles of dormancy and growth in pea axillary buds based on mRNA accumulation patterns of cell cycle-related genes. Plant and Cell Physiology 39, 255–262. 9588023 10.1093/oxfordjournals.pcp.a029365

[CIT0041] Shimizu S Mori H 1998 *b* Changes in protein interactions of cell cycle-related genes during the dormancy-to-growth transition in pea axillary buds. Plant and Cell Physiology 39, 1073–1079. 9871367 10.1093/oxfordjournals.pcp.a029304

[CIT0042] Swofford DL 2003 PAUP*. Phylogenetic Analysis Using Parsimony (* and Other Methods) Version 4. Sinauer Associates, Sunderland.

[CIT0043] Takei K Sakakibara H Taniguchi M Sugiyama T 2001 Nitrogen-dependent accumulation of cytokinins in root and the translocation to leaf: implication of cytokinin species that induces gene expression of maize response regulator. Plant and Cell Physiology 42, 85–93. 11158447 10.1093/pcp/pce009

[CIT0044] Uggla C Magel E Moritz T Sundberg B 2001 Function and dynamics of auxin and carbohydrates during earlywood/latewood transition in Scots pine. Plant Physiology 125, 2029–2039. 11299382 10.1104/pp.125.4.2029 PMC88858

[CIT0045] Uggla C Mellerowicz EJ Sundberg B 1998 Indole-3-acetic acid controls cambial growth in Scots pine by positional signaling. Plant Physiology 117, 113–121. 9576780 10.1104/pp.117.1.113 PMC34994

[CIT0046] Uggla C Moritz T Sandberg G Sundberg B 1996 Auxin as a positional signal in pattern formation in plants. Proceedings of the National Academy of Sciences, USA 93, 9282–9286. 10.1073/pnas.93.17.9282 PMC38633 11607701

[CIT0047] Villordon AQ Ginzberg I Firon N 2014 Root architecture and root and tuber crop productivity. Trends in Plant Science 19, 419–425. 24630073 10.1016/j.tplants.2014.02.002

[CIT0048] Webster BD Radin JW 1972 Growth and development of cultured radish roots. American Journal of Botany 59, 744–751.

[CIT0049] Xu Y Zhu X Gong Y Xu L Wang Y Liu L 2012 Evaluation of reference genes for gene expression studies in radish (*Raphanus sativus* L.) using quantitative real-time PCR. Biochemical and Biophysical Research Communications 424, 398–403. 22771808 10.1016/j.bbrc.2012.06.119

[CIT0050] Yang J Zhang J Wang Z Zhu Q Wang W 2001 Hormonal changes in the grains of rice subjected to water stress during grain filling. Plant Physiology 127, 315–323. 11553759 10.1104/pp.127.1.315 PMC117987

[CIT0051] Yordanov YS Regan S Busov V 2010 Members of the LATERAL ORGAN BOUNDARIES DOMAIN transcription factor family are involved in the regulation of secondary growth in *Populus* . The Plant Cell 22, 3662–3677. 21097711 10.1105/tpc.110.078634 PMC3015109

[CIT0052] Zhang J Elo A Helariutta Y 2011 *Arabidopsis* as a model for wood formation. Current Opinion in Biotechnology 22, 293–299. 21144727 10.1016/j.copbio.2010.11.008

[CIT0053] Zhang J Nieminen K Serra JAA Helariutta Y 2014 The formation of wood and its control. Current Opinion in Plant Biology 17, 56–63. 24507495 10.1016/j.pbi.2013.11.003

