# Supplementary material for: Cytokinin-dependent secondary growth determines root biomass in radish (Raphanus sativus L.)
Source: J Exp Bot. 2015 May 15;66(15):4607–19. doi: 10.1093/jxb/erv220 (PMC4507762; doi:10.1093/jxb/erv220)
Supplement: Supplementary Data [file supp_66_15_4607__index.html]

Cytokinin-dependent secondary growth determines root biomass in radish (Raphanus sativus L.) — Cytokinin-dependent secondary growth determines root biomass in radish (Raphanus sativus L.) — Supplementary Data 

# Cytokinin-dependent secondary growth determines root biomass in radish (*Raphanus sativus* L.)

## Supplementary Data

Data files

**Files in this Data Supplement:**

- Supplementary Data - Supplementary Data
