# Supplementary material for: Cytokinin-dependent secondary growth determines root biomass in radish (Raphanus sativus L.)
Source: J Exp Bot. 2015 May 15;66(15):4607–19. doi: 10.1093/jxb/erv220 (PMC4507762; doi:10.1093/jxb/erv220)
Supplement: Supplementary Data [file supp_erv220_jexbot143131_file001.pdf]

Supplementary Data

Title: Cytokinin-dependent secondary growth determines root biomass in radish (*Raphanus sativus* L.)

Geupil Jang[1], Jung-Hun Lee[1], Khushboo Rastogi[1], Suhyoung Park[2], Sang-Hun Oh[3], Ji-Young Lee[1*]

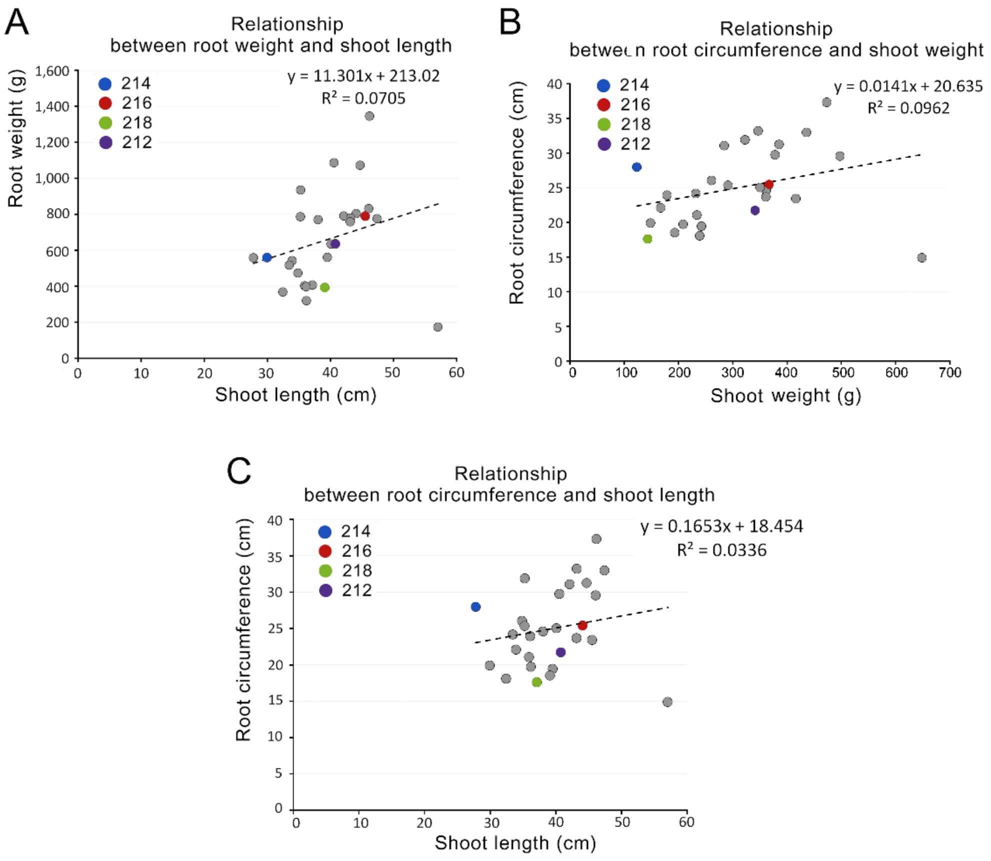

Figure S1. Relationships between radish growth factors.

A-C) Scatter plots showing relationships between root weight and shoot length (**A**), between root circumference and shoot weight (**B**) and between root circumference and shoot length (**C**). Dotted lines indicate trend lines.

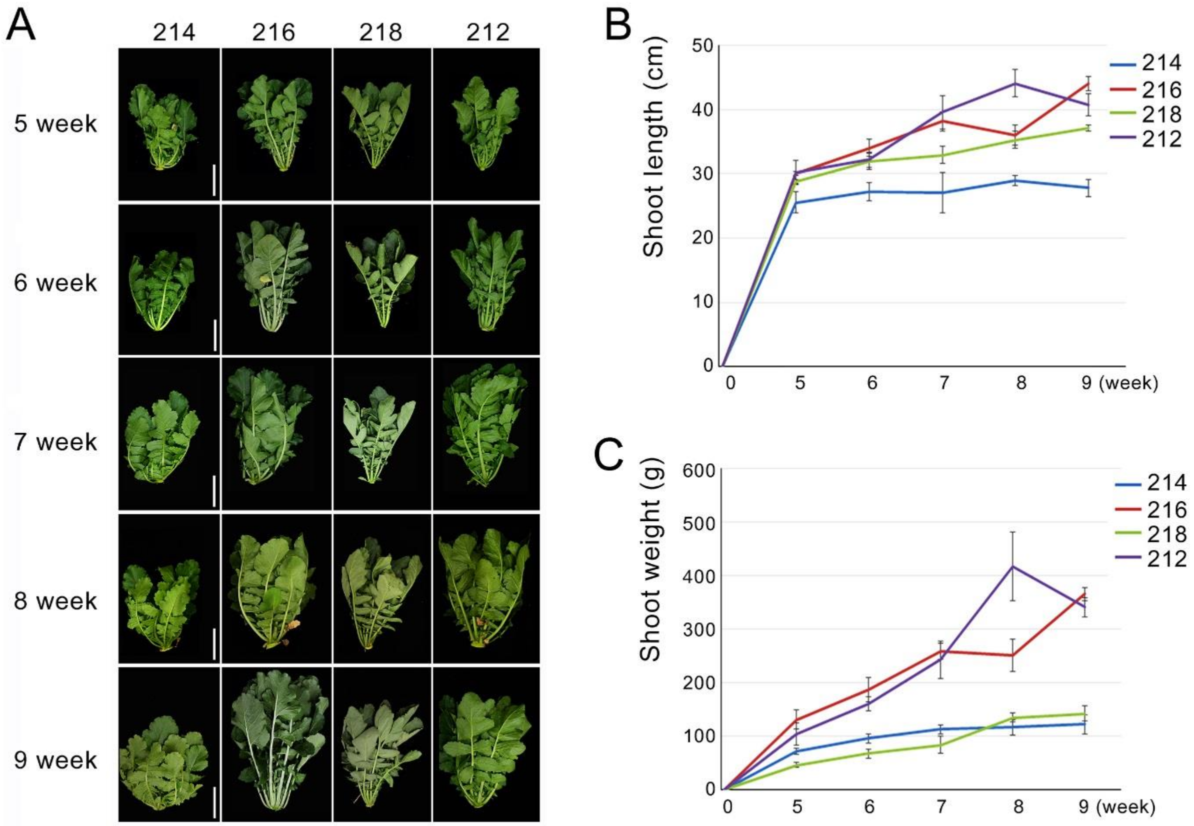

Figure S2. Shoot growth pattern in selected radish inbred lines.

A) Time-course shoot images of radish inbred lines, 214, 216, 218 and 212.

B and C) Shoot growth patterns of radish inbred lines. Growth patterns of the indicated inbred lines were analyzed by measuring shoot length **(B)** and shoot weight **(C)** at the indicated time (n≥4). Scale bars = 10 cm.

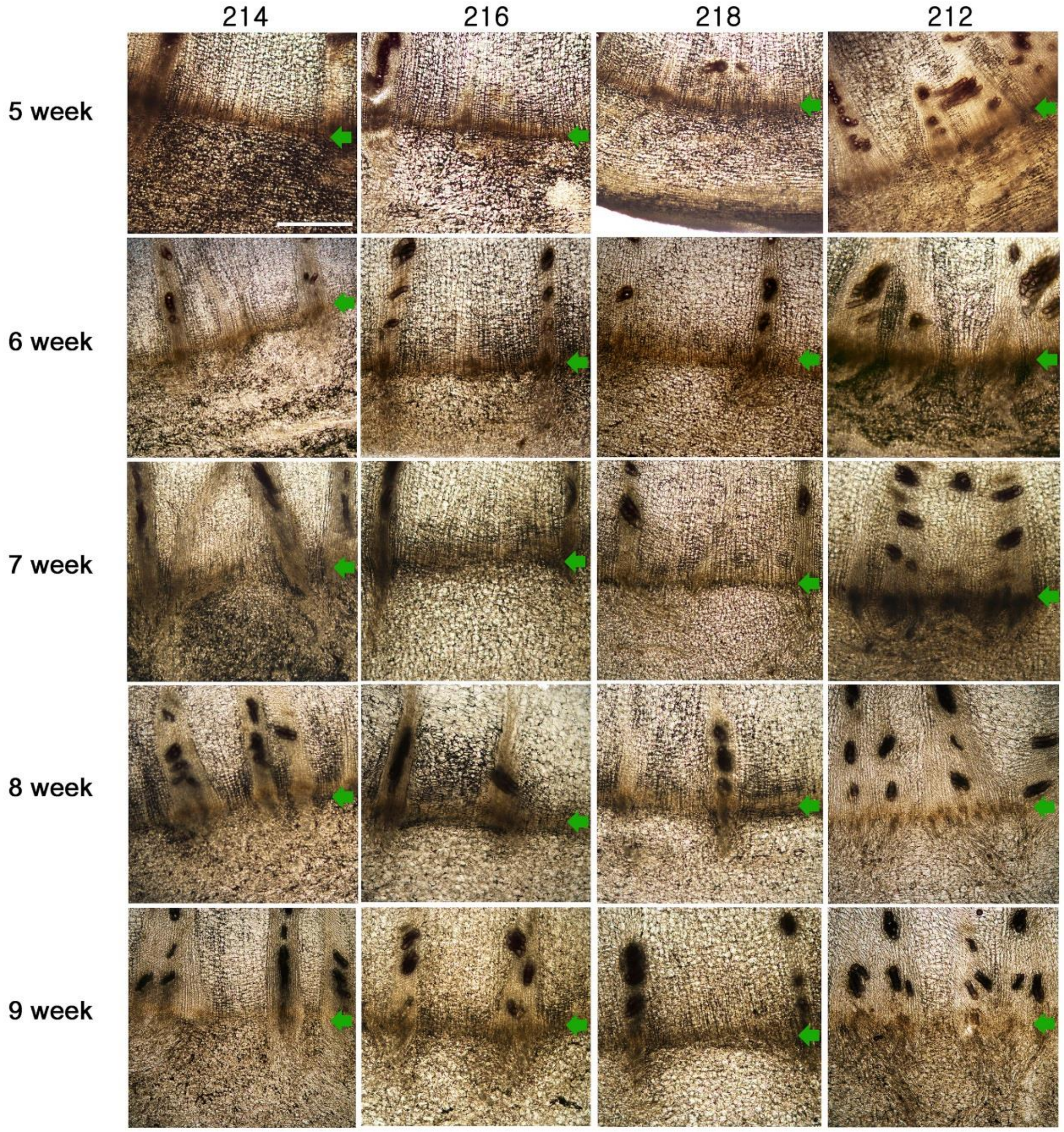

Figure S3. Images of root cross-sections in selected radish inbred lines.

Analysis of internal root anatomy of radish inbred lines along developmental stages. Green arrows indicate cambium regions. Scale bars = 1 mm.

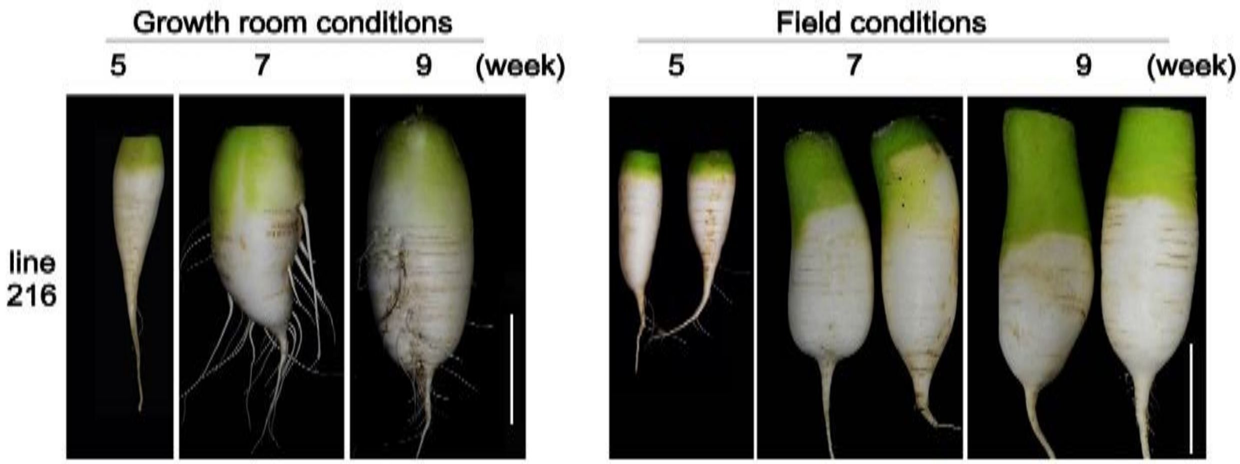

Figure S4. Radish root development in growth room and field conditions.

Morphology of line 216 roots grown in growth room (left) and field conditions (right) for 5, 7 and 9 week. Scale bars = 10 cm.

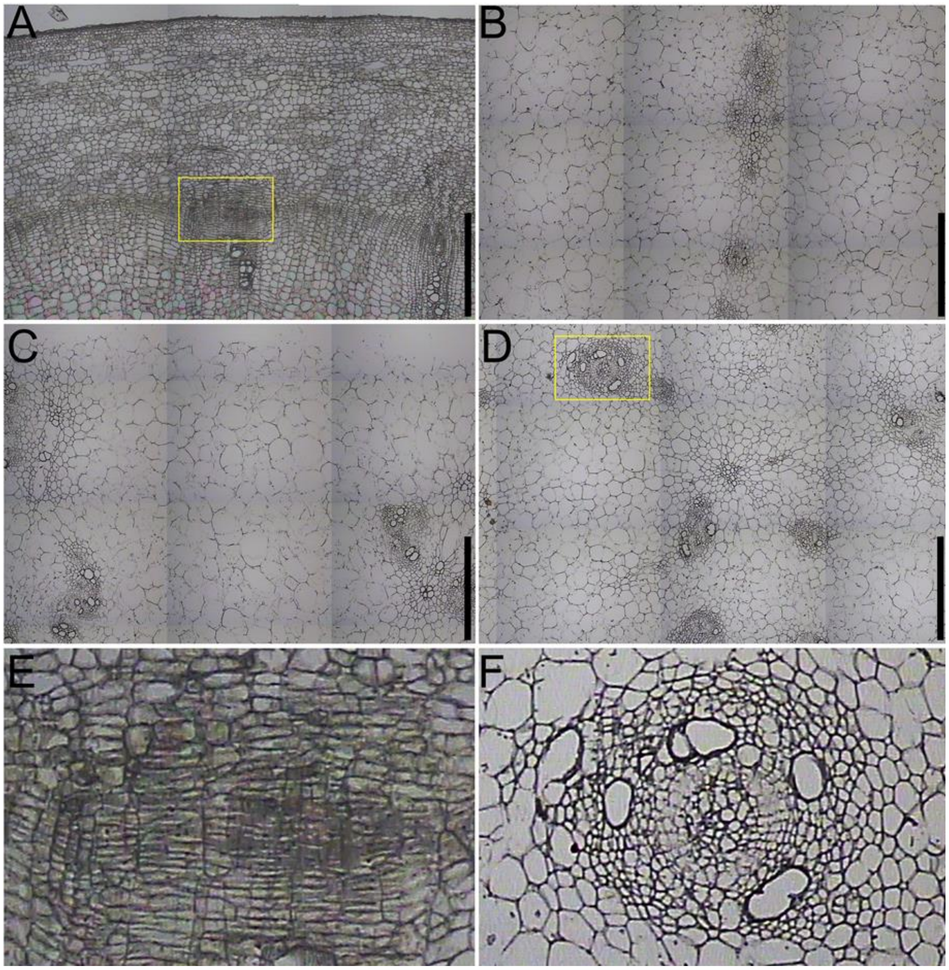

Figure S5. Internal anatomy of radish roots.

Transverse sectioning images showing cortex and cambium region **(A)**, inner parenchyma region 1 **(B)**, inner parenchyma region 2 **(C)** and central region **(D)**. **E)** High magnification of cambium zone (yellow box) of **(A). F)** High magnification of ectopic meristem region (yellow box) of **(D)**. Scale bar = 500 um.

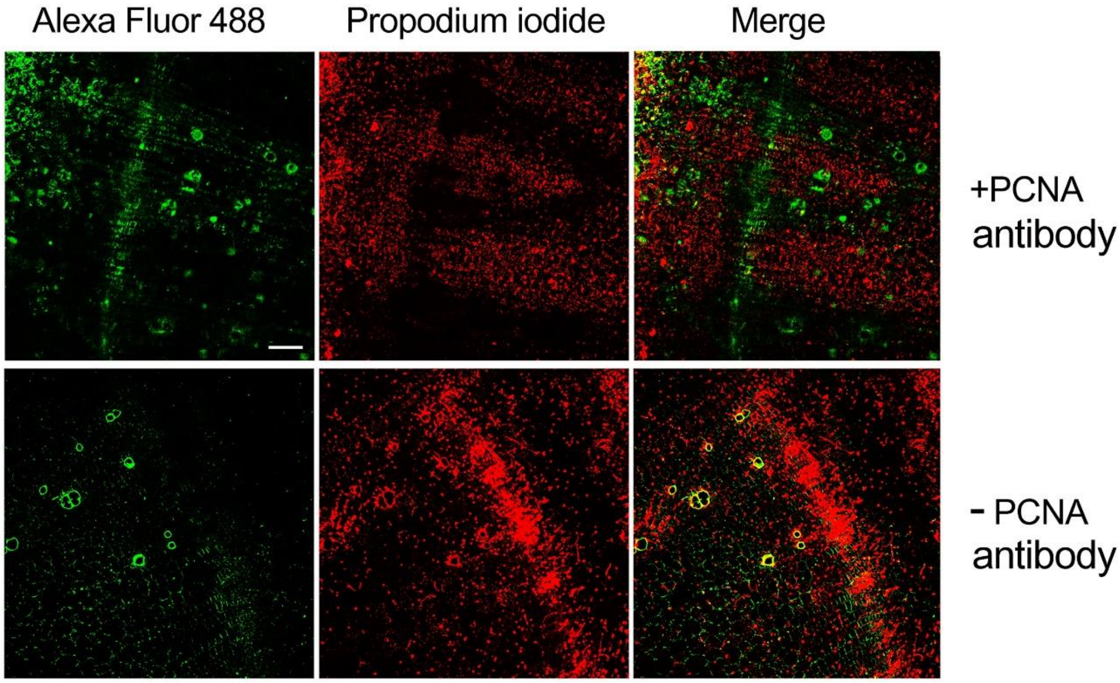

Figure S6. Cell division activities in root cambia.

Dividing cell-specific expression of PCNA was visualized by immunolocalization using 1-month-old scarlet globe roots. Alexa Fluor® 488 Anti-Goat IgG signal is observed in the cambium in the tissue without PCNA antibody treatment. Scale bars = 200 μm.

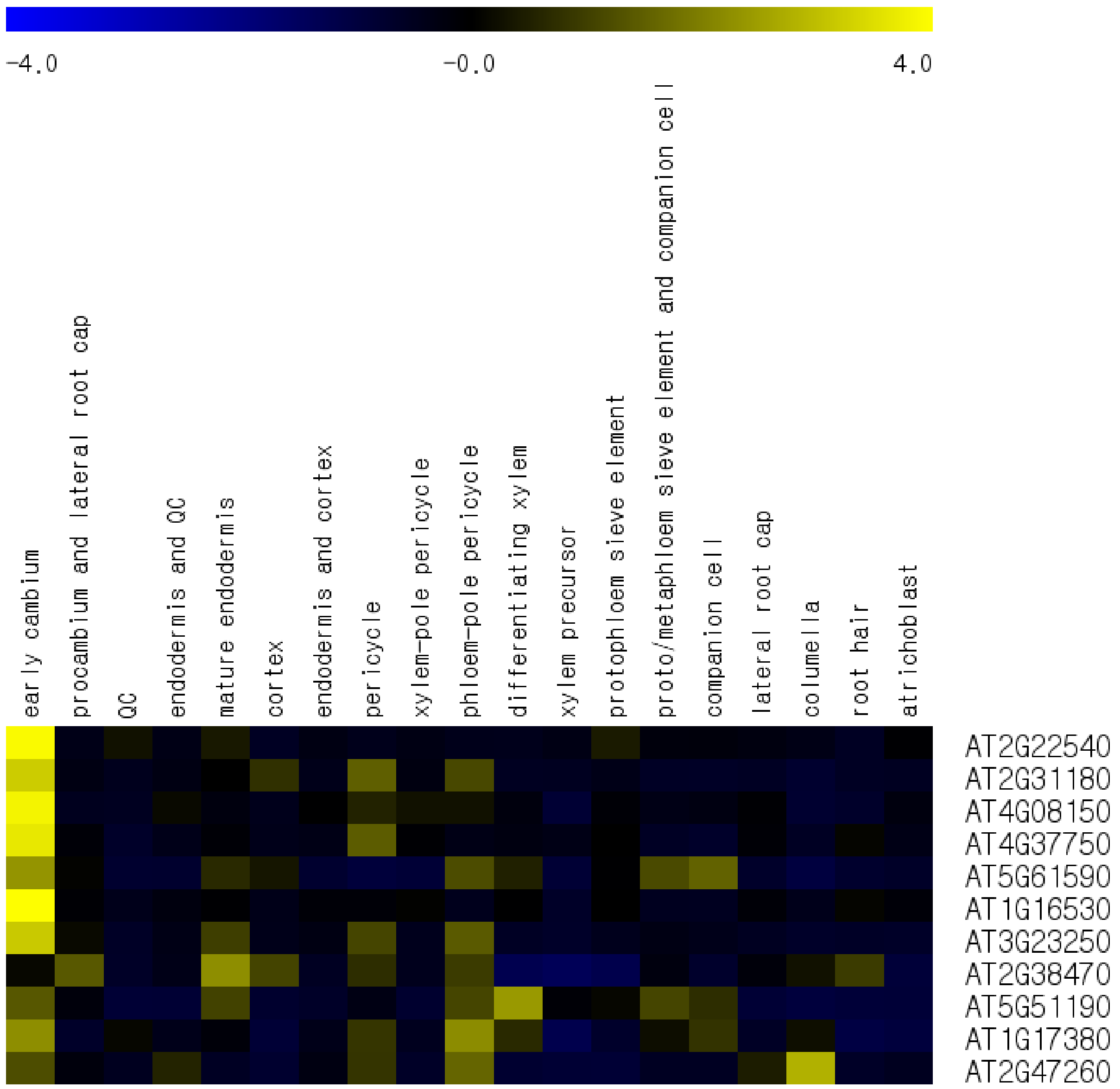

Figure S7. Cambium-enriched transcription factor genes in *Arabidopsis*.

A heat map showing expression patterns of cambium-enriched transcription factor genes in *Arabidopsis* root. Gene expression was normalized by each row.

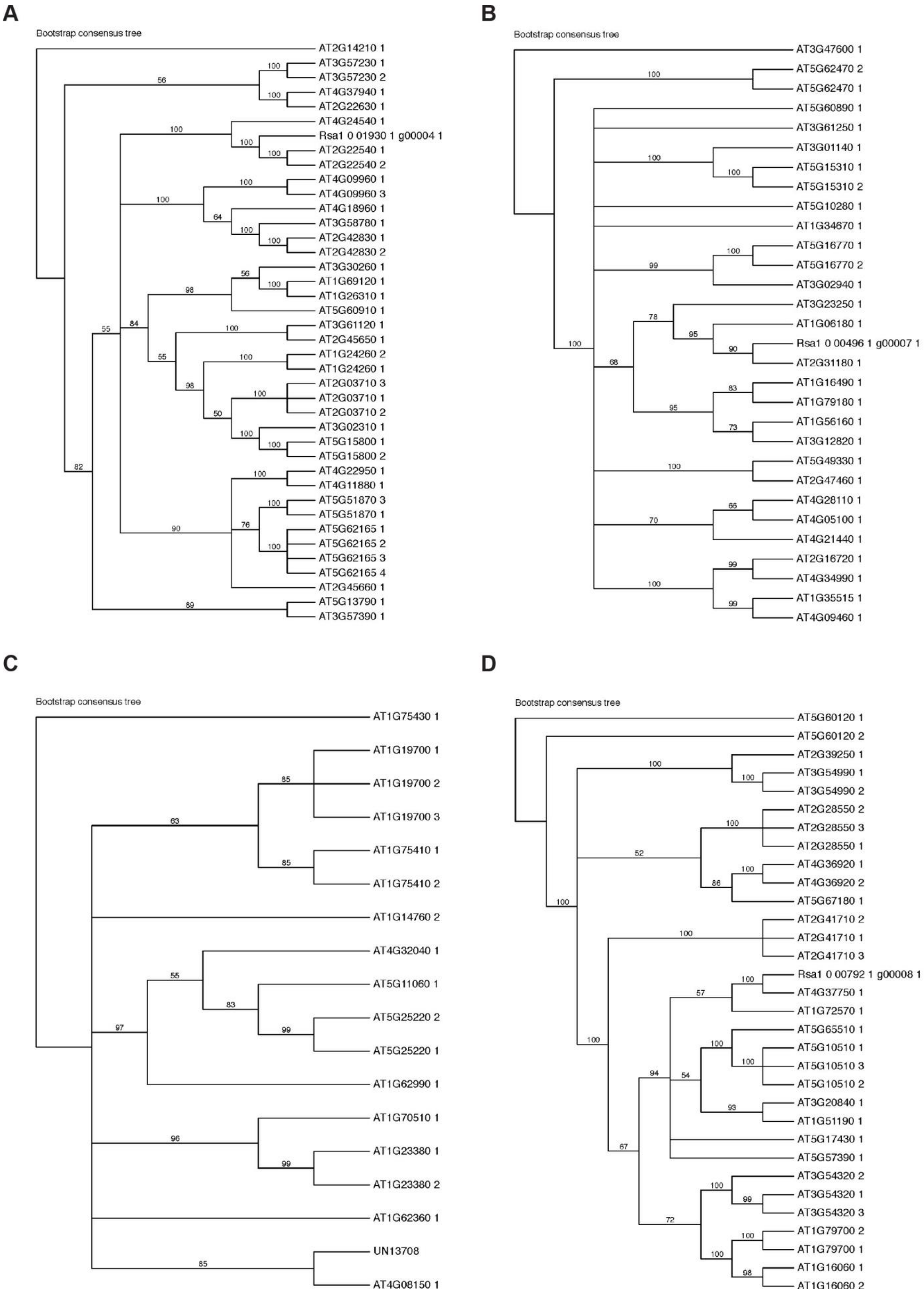
A
Bootstrap consensus tree
AT2G14210 1
AT3G57230 1
AT3G57230 2
AT4G37940 1
AT2G22630 1
AT4G24540 1
Rsa1 0 01930 1 g00004 1
AT2G22540 1
AT2G22540 2
AT4G09960 1
AT4G09960 3
AT4G18960 1
AT3G58780 1
AT2G42830 1
AT2G42830 2
AT3G30260 1
AT1G69120 1
AT1G26310 1
AT5G60910 1
AT3G61120 1
AT2G45650 1
AT1G24260 2
AT1G24260 1
AT2G03710 3
AT2G03710 1
AT2G03710 2
AT3G02310 1
AT5G15800 1
AT5G15800 2
AT4G22950 1
AT4G11880 1
AT5G51870 3
AT5G51870 1
AT5G62165 1
AT5G62165 2
AT5G62165 3
AT5G62165 4
AT2G45660 1
AT5G13790 1
AT3G57390 1
B
Bootstrap consensus tree
AT3G47600 1
AT5G62470 2
AT5G62470 1
AT5G60890 1
AT3G61250 1
AT3G01140 1
AT5G15310 1
AT5G15310 2
AT5G10280 1
AT1G34670 1
AT5G16770 1
AT5G16770 2
AT3G02940 1
AT3G23250 1
AT1G06180 1
Rsa1 0 00496 1 g00007 1
AT2G31180 1
AT1G16490 1
AT1G79180 1
AT1G56160 1
AT3G12820 1
AT5G49330 1
AT2G47460 1
AT4G28110 1
AT4G05100 1
AT4G21440 1
AT2G16720 1
AT4G34990 1
AT1G35515 1
AT4G09460 1
C
Bootstrap consensus tree
AT1G75430 1
AT1G19700 1
AT1G19700 2
AT1G19700 3
AT1G75410 1
AT1G75410 2
AT1G14760 2
AT4G32040 1
AT5G11060 1
AT5G25220 2
AT5G25220 1
AT1G62990 1
AT1G70510 1
AT1G23380 1
AT1G23380 2
AT1G62360 1
UN13708
AT4G08150 1
D
Bootstrap consensus tree
AT5G60120 1
AT5G60120 2
AT2G39250 1
AT3G54990 1
AT3G54990 2
AT2G28550 2
AT2G28550 3
AT2G28550 1
AT4G36920 1
AT4G36920 2
AT5G67180 1
AT2G41710 2
AT2G41710 1
AT2G41710 3
Rsa1 0 00792 1 g00008 1
AT4G37750 1
AT1G72570 1
AT5G65510 1
AT5G10510 1
AT5G10510 3
AT5G10510 2
AT3G20840 1
AT1G51190 1
AT5G17430 1
AT5G57390 1
AT3G54320 2
AT3G54320 1
AT3G54320 3
AT1G79700 2
AT1G79700 1
AT1G16060 1
AT1G16060 2

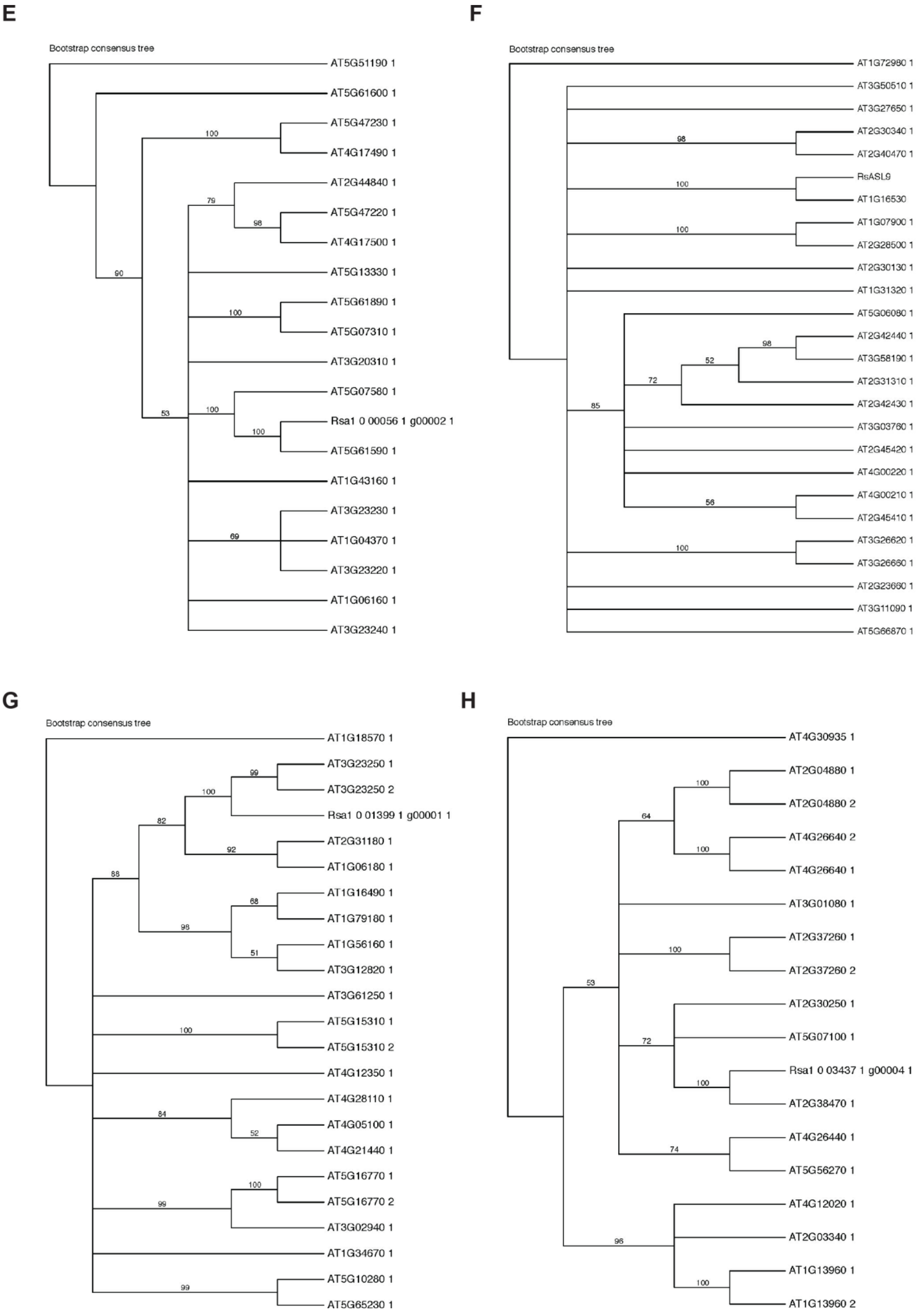
E
Bootstrap consensus tree
AT5G51190 1
AT5G61600 1
AT5G47230 1
AT4G17490 1
AT2G44840 1
AT5G47220 1
AT4G17500 1
AT5G13330 1
AT5G61890 1
AT5G07310 1
AT3G20310 1
AT5G07580 1
Rsa1 0 00056 1 g00002 1
AT5G61590 1
AT1G43160 1
AT3G23230 1
AT1G04370 1
AT3G23220 1
AT1G06160 1
AT3G23240 1
100
79
98
90
100
53
100
100
69
F
Bootstrap consensus tree
AT1G72980 1
AT3G50510 1
AT3G27650 1
AT2G30340 1
AT2G40470 1
RsASL9
AT1G16530
AT1G07900 1
AT2G28500 1
AT2G30130 1
AT1G31320 1
AT5G06080 1
AT2G42440 1
AT3G58190 1
AT2G31310 1
AT2G42430 1
AT3G03760 1
AT2G45420 1
AT4G00220 1
AT4G00210 1
AT2G45410 1
AT3G26620 1
AT3G26660 1
AT2G23660 1
AT3G11090 1
AT5G66870 1
98
100
100
98
52
72
85
56
100
G
Bootstrap consensus tree
AT1G18570 1
AT3G23250 1
AT3G23250 2
Rsa1 0 01399 1 g00001 1
AT2G31180 1
AT1G06180 1
AT1G16490 1
AT1G79180 1
AT1G56160 1
AT3G12820 1
AT3G61250 1
AT5G15310 1
AT5G15310 2
AT4G12350 1
AT4G28110 1
AT4G05100 1
AT4G21440 1
AT5G16770 1
AT5G16770 2
AT3G02940 1
AT1G34670 1
AT5G10280 1
AT5G65230 1
99
100
82
92
88
68
98
51
100
84
52
100
99
99
H
Bootstrap consensus tree
AT4G30935 1
AT2G04880 1
AT2G04880 2
AT4G26640 2
AT4G26640 1
AT3G01080 1
AT2G37260 1
AT2G37260 2
AT2G30250 1
AT5G07100 1
Rsa1 0 03437 1 g00004 1
AT2G38470 1
AT4G26440 1
AT5G56270 1
AT4G12020 1
AT2G03340 1
AT1G13960 1
AT1G13960 2
100
64
100
100
53
72
100
74
96
100

I

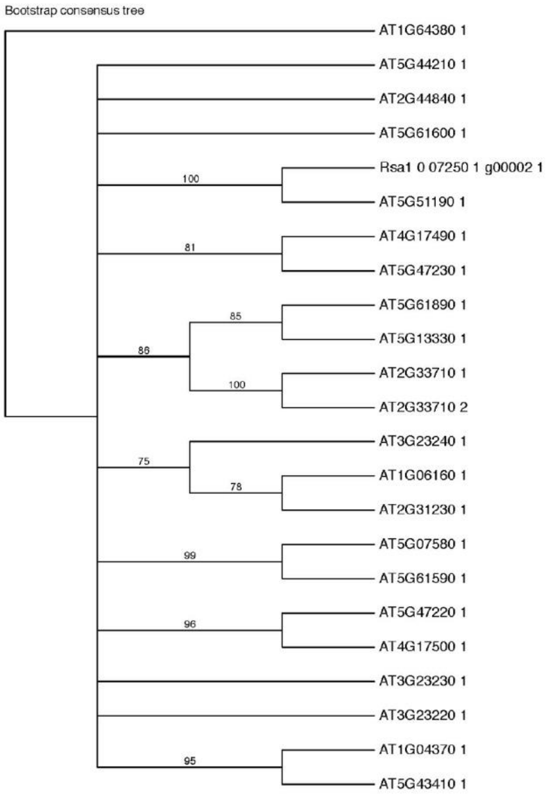
Bootstrap consensus tree
AT1G64380 1
AT5G44210 1
AT2G44840 1
AT5G61600 1
Rsa1 0 07250 1 g00002 1
AT5G51190 1
AT4G17490 1
AT5G47230 1
AT5G61890 1
AT5G13330 1
AT2G33710 1
AT2G33710 2
AT3G23240 1
AT1G06160 1
AT2G31230 1
AT5G07580 1
AT5G61590 1
AT5G47220 1
AT4G17500 1
AT3G23230 1
AT3G23220 1
AT1G04370 1
AT5G43410 1
100
81
85
86
100
75
78
99
96
95

J

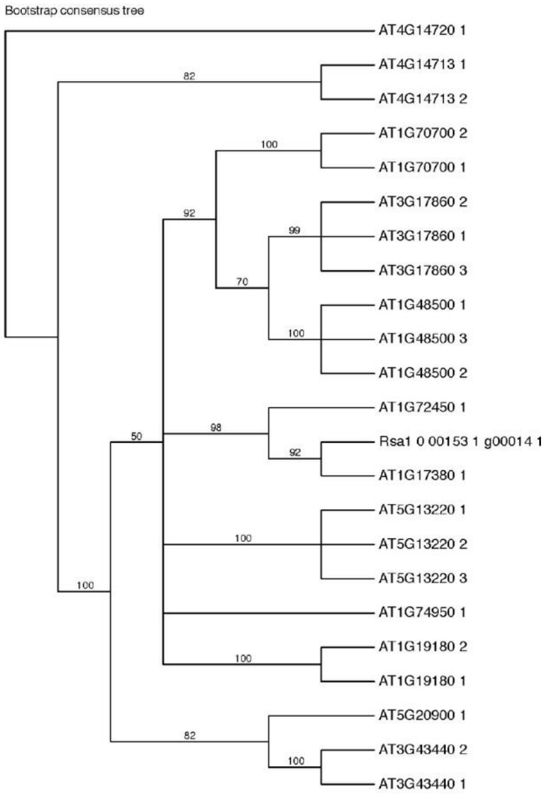
Bootstrap consensus tree
AT4G14720 1
AT4G14713 1
AT4G14713 2
AT1G70700 2
AT1G70700 1
AT3G17860 2
AT3G17860 1
AT3G17860 3
AT1G48500 1
AT1G48500 3
AT1G48500 2
AT1G72450 1
Rsa1 0 00153 1 g00014 1
AT1G17380 1
AT5G13220 1
AT5G13220 2
AT5G13220 3
AT1G74950 1
AT1G19180 2
AT1G19180 1
AT5G20900 1
AT3G43440 2
AT3G43440 1
82
100
92
99
70
100
50
98
92
100
100
100
82
100

K

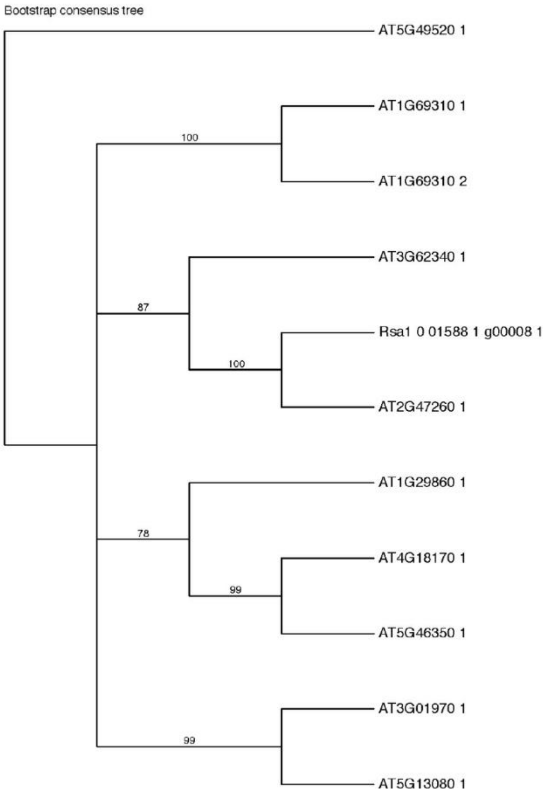
Bootstrap consensus tree
AT5G49520 1
AT1G69310 1
AT1G69310 2
AT3G62340 1
Rsa1 0 01588 1 g00008 1
AT2G47260 1
AT1G29860 1
AT4G18170 1
AT5G46350 1
AT3G01970 1
AT5G13080 1
100
87
100
78
99
99

Figure S8. Phylogenetic analyses of cambium-enriched transcription factor genes in Arabidopsis and their putative radish orthologs.

Bootstrap consensus trees for **A)** AT2G22540, **B)** AT2G31180, **C)** AT4G08150, **D)** AT3G37750, **E)** AT5G61590, **F)** AT1G16530, **G)** AT3G23250, **H)** AT2G38470, **I)** AT5G51190, **J)** AT1G17380, and **K)** AT2G47260. Putative radish CDS were used for phylogenetic analyses except for AT4G08150.

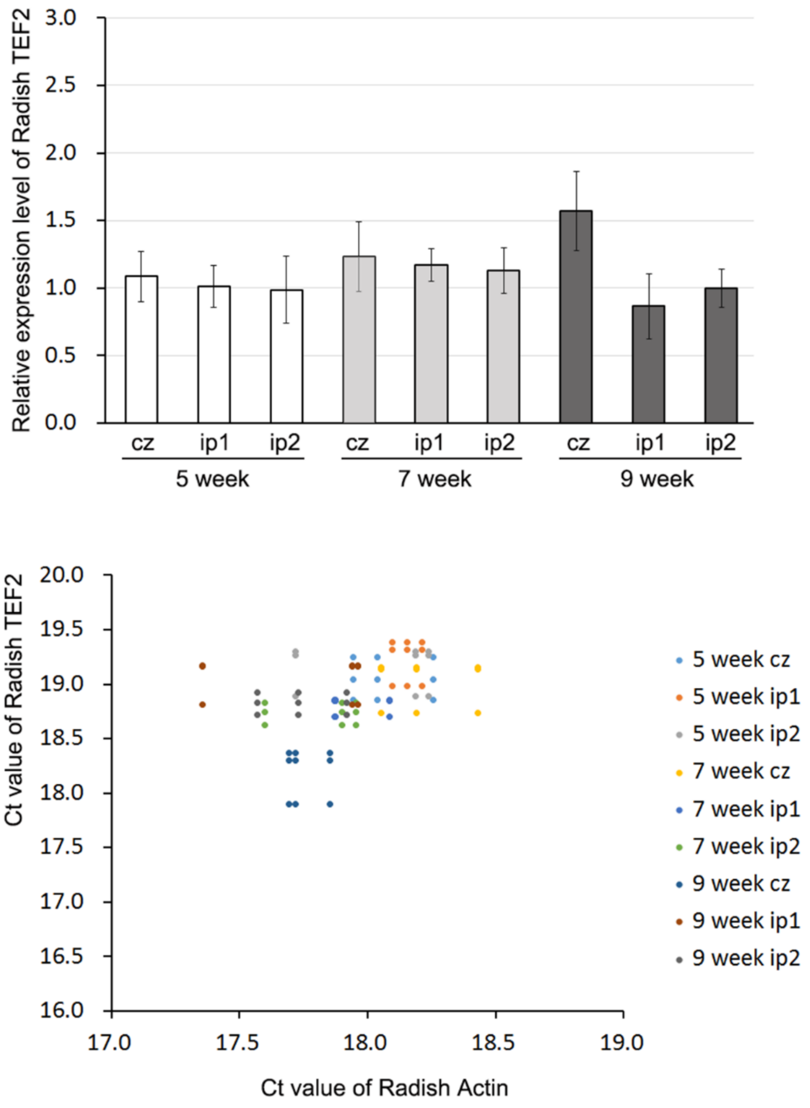

Figure S9. Validation of Actin2/7 expression as a reference gene for quantitative RT-PCR analysis in radish. Top) Relative expression of TEF2, a radish reference gene in cambium zone, inner parenchyma region 1 and region 2 of line 216 against radish Actin2/7 genes. **Bottom**) A scatter plot using Ct values of TEF2 and Actin2/7.

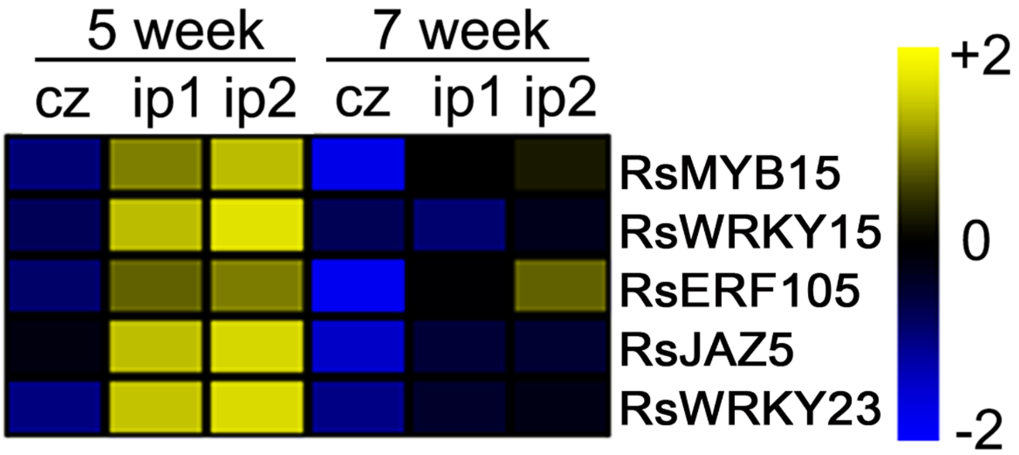

Figure S10. Expression patterns of the radish candidates.

A heap map showing expression patterns of RsMYB15, RsWRKY15, RsERF105, RsJAZ5 and RsWRKY23 in five-and seven-week-old line 216 roots. Gene expression was normalized by each row.

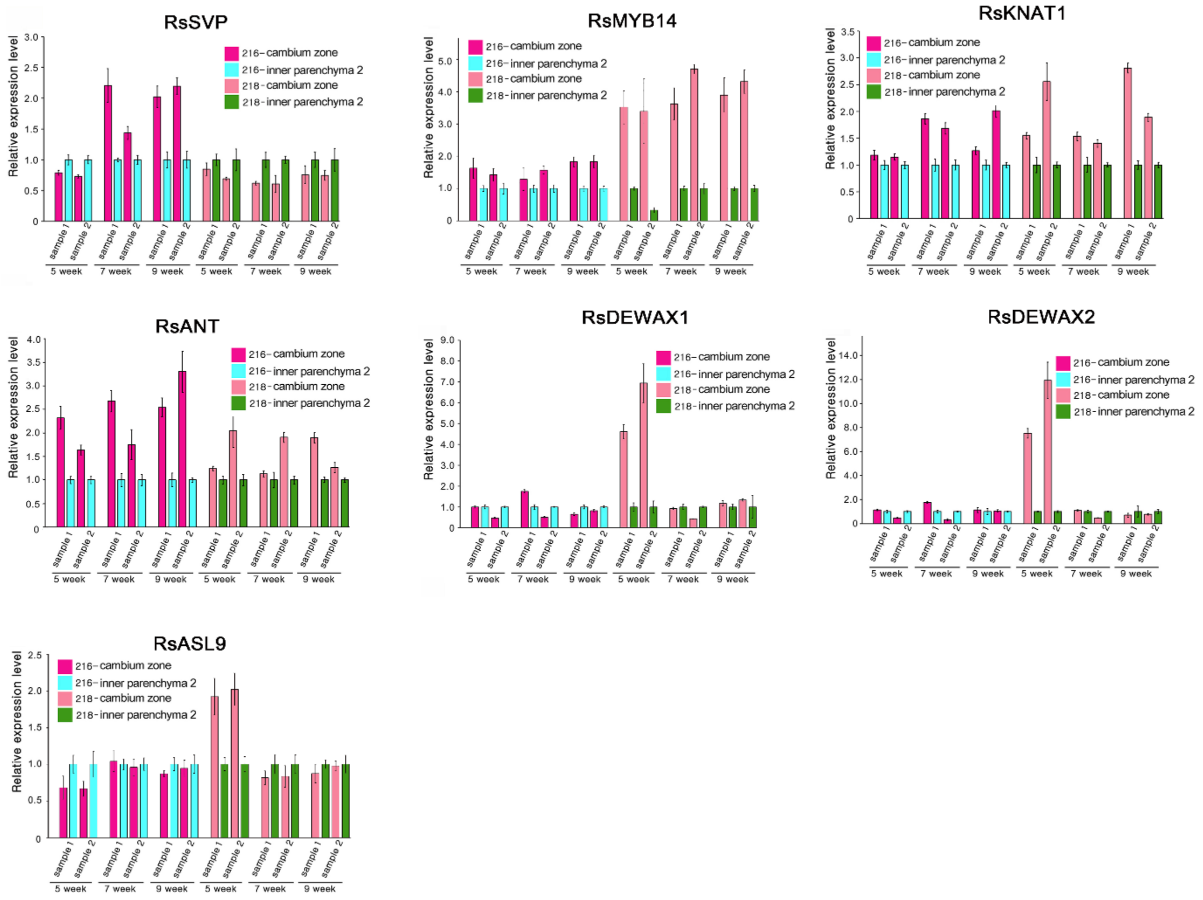

Figure S11. Distinct expression pattern of the radish candidates between lines 216 and 218.

Distinct expression pattern of the candidate genes between line 216 and 218 was analyzed by quantitative RT-PCR analysis. Gene expression in inner parenchyma region 2 was used as normalization control for analyzing relative expression of the candidate gene in cambium zone.

Table S1. Primers used in this study

Primer name	Sequences
RsSVP_for	GCATGAAAGATACGGTGGAG
RsSVP _rev	GTATCGGAGCTCTCGGAGT
RsMYB14_for	CAAAACAATGGCGGAGACAC
RsMYB14_rev	CTGTTGCGGAGAGGTTGTGT
RsKNAT1_for	CCGAAGACATGCAGTTTATGG
RsKNAT1_rev	GGCTAACCCTTCTGGATATC
RsANT_for	GGCCGTTTTTCTTACATTGCC
RsANT _rev	CCGGATAAAACCGTTCATAAAC
RsDEWAX1_for	CGAACTAGGGATTGGTGATG
RsDEWAX1_rev	GTTCCATTGTCACAGGAACTC
RsDEWAX2_for	GGAGAAGAAACGATGTAACGG
RsDEWAX2_rev	GGCTCTATACATGATGCTTCA
RsASL9_for	CAAACTCAGCTCGCTTTTGC
RsASL9_rev	CTAATGCCACTCGCCATGTA
RsMYB15_for	CAACGCGTCGAATCCTGAAGT
RsMYB15_rev	GCCAATACATCGAGCCAGAAG
RsWRKY33_for	GAGCACCATATACGCTTCAGA
RsWRKY33_rev	GAGAATCCACCAACAAACTCG
RsERF105_for	CTTAACTTCCCACTCGAAGCA
RsERF105_rev	GGTTGATCAGGTTTATCCCATG
RsJAZ5_for	GTACCTGGTCAACAGCTAGA
RsJAZ5_rev	GCCTAAGTTCGAGATCTTTCG
RsWRKY 23_for	GTCTTGTTTTGGAGGAGTCG
RsWRKY 23_rev	CCTTCAACATATGACACGGG
RsRR15_for	GATGATGAAACTCCATCTTCC
RsRR15_rev	GATCTTAGCACAACATCTTGG

RsRR7_for	GGAGAAGATGCTGATACATCA
RsRR7_rev	GGTCTTGGCTCTATGTATTTC
RsActin2/7_for	GCATCACACTTTCTACAAC
RsActin2/7_rev	CCTGGATAGCAACATACAT
RsTEF2_for	AAGAAGATTTGGGCGTTTGG
RsTEF2_rev	CCATCAACAACAGAATCCTT

Table S2. Quantification of cambial cell layers in line 216 and 218

Average number of cambial cell layers	5 week	7 week	9 week
**Line 216**	11.976	13.763	5.324
**Line 218**	5.064	5.523	4.420
**P value**	0.0001>	0.0001>	0.0013
